# Tear biomarker changes and ocular surface recovery with low-level light therapy after cataract surgery: a double-masked randomized controlled clinical trial

**DOI:** 10.1038/s41598-026-53521-4

**Published:** 2026-05-20

**Authors:** Mihaela-Madalina Timofte-Zorila, Mariana Pavel-Tanasa, Giuseppe Giannaccare, Nicoleta Vlas, Filippo Lixi, Mario Troisi, Daniela Constantinescu, Radu Tanasa, Diana Alecu, Sabina Turcas, Sinziana Istrate, Daciana Elena Branisteanu, Cristina Preda, Daniel Constantin Branisteanu

**Affiliations:** 1 Grigore T. Popa University of Medicine and Pharmacy Iasi, 700115 Iasi, Romania; 2 Ophthalmology Clinic, Cai Ferate Clinical Hospital, 700506 Iasi, Romania; 3 Laboratory of Immunology, St. Spiridon County Clinical Emergency Hospital, 700111 Iasi, Romania; 4 Department of Surgical Sciences, Eye Clinic, University of Cagliari, 09124 Cagliari, Italy; 5 Department of Neurosciences, Reproductive and Odontostomatological Sciences, Eye Clinic, Federico II University, 80131 Naples, Italy; 6 Ophthalmologic Unit, Salerno University Hospital, 84131 Salerno, Italy; 7 Faculty of Physics, Alexandru Ioan Cuza University, 700506 Iasi, Romania; 8 Carol Davila University of Medicine and Pharmacy, 050474 Bucharest, Romania; 9 Optissima, Barbu Văcărescu 102, 020283 Bucharest, Romania; 10 Dermatology Clinic, Cai Ferate Clinical Hospital, 700506 Iasi, Romania

**Keywords:** Low-level light therapy, Dry eye disease, Cataract surgery, GDF-15, β-NGF, PDGF-CC, OPN, OSDI, TBUT, Schirmer test, Biomarkers, Diseases, Medical research

## Abstract

Dry eye disease (DED) is a common complication following cataract-surgery, potentially impairing visual recovery. Low-level light therapy (LLLT) has emerged as a noninvasive approach to improve ocular surface status. This study evaluated the perioperative effects of LLLT on ocular surface modulation by correlating clinical outcomes with tear biomarker dynamics and developing predictive models to identify patients most likely to benefit from LLLT. Of the 98 patients initially randomized, 88 were included in the final analysis, with 44 allocated to the LLLT group and 44 to the sham treatment group. Clinical evaluation—including Ocular Surface Disease Index (OSDI) as the primary outcome, and tear breakup time, Schirmer test, and tear osmolarity as secondary endpoints—was performed preoperatively and one month postoperatively to classify patients as preclinical or having DED. Tear levels of biomarkers involved in tissue repair and neurotrophic-signaling (GDF-15,β-NGF, VEGFA, PDGF-AB, PDGF-CC) and inflammation (OPN, OPG, TNF-α) were assessed as exploratory endpoints and quantified using Luminex-FlexMap3D technology. LLLT-treated patients showed significantly higher clinical improvement post-cataract surgery than sham controls (44.2% vs. 4.4%; *p* < 0.0001). In DED patients, LLLT significantly increased GDF-15 (77.6 ± 7.2 to 95.5 ± 7.4 pg/mL; *p* = 0.0112) and PDGF-CC (711.4 ± 76.6 to 1024 ± 130.8 pg/mL; *p* = 0.0473). The LLLT-induced β-NGF increase was more pronounced in preclinical cases than in those with baseline DED (10.1 ± 3.02 vs. 5.8 ± 0.55 pg/mL; *p* = 0.0271). Biomarker profiles indicated inflammatory resolution post-LLLT, whereas sham-treated eyes showed persistent inflammation. Finally, integrative modeling of clinical and molecular data yielded a LASSO-adjusted AUC of 0.886, underscoring high discriminative performance. LLLT accelerates ocular surface recovery after cataract surgery by modulating reparative, neurotrophic, and inflammatory pathways. Tear biomarkers assessed at baseline and through one-month dynamics (GDF-15, PDGF-CC, β-NGF) and integrated with clinical parameters, may help predict treatment response and guide personalized postoperative management.

**Supplementary Information:**

The online version contains supplementary material available at 10.1038/s41598-026-53521-4.

## Introduction

Nowadays, cataract surgery is one of the most frequently performed surgical procedures worldwide, with excellent visual outcomes in the vast majority of cases. Despite its favorable prognosis in terms of clinical results and postoperative recovery, cataract surgery may be associated with several complications, among which postoperative dry eye disease (DED) is one of the most common^1^. Although there is still a lack of consensus on the actual prevalence and duration of DED following cataract surgery, this association is known to be clinically relevant, multifactorial and complex^2^. The ocular surface alterations are inflammatory, structural, and functional as the result of combined mechanisms of corneal nerve transection and other intraoperative injuries, inflammatory cascade with the release of cytokines and free radicals, loss of goblet cells, meibomian gland dysfunction (MGD), and the use of preserved eye drops^3^. Regarding the inflammatory biomarkers, there is a significant correlation between DED and the increase in their expression of various cytokines, such as tumor necrosis factor alpha (TNF-α), interleukin 6 (IL-6), IL-8, and IL-17, matrix metalloproteinase-9 (MMP-9), Interferon gamma (IFN-γ) among others^4^.

In-office instrumental therapies represent a new option in the armamentarium of DED treatment. Among these, low-level light therapy (LLLT), also termed photobiomodulation or phototherapy, has recently gained increased attention for both managing and preventing DED in the setting of ocular surgery^5^. This therapy is based on the use of various nonionizing forms of light in the visible and near-infrared (NIR) spectrum from sources like lasers, light-emitting diodes (LED), and filtered lamps able to activate a cascade of complex photophysical and photochemical events at various biological scales^5,6^. These molecular events result in cellular responses that promote migration, proliferation, anti-inflammatory activity, and tissue repair via transcription factor activation and regulation of cytokines and growth factors^7–10^.

The aim of the study was to investigate the dynamics of clinical outcomes, with OSDI as the primary endpoint, and additional ocular surface parameters (including tear film breakup time (TBUT), corneal fluorescein staining, the Schirmer I test, and tear osmolarity) as secondary, in relation to tear film inflammatory and reparative biomarkers assessed as exploratory endpoints, and to identify associations between these clinical and biological measures with potential predictive value. This integrated clinical–molecular approach may help identify prognostic factors capable of predicting which patients are most likely to benefit from LLLT and to determine whether it provides superior modulation of ocular surface status compared to standard postoperative care alone, particularly in terms of inflammatory regulation, neurotrophic support, and tissue repair.

## Methods

### Study design and participants

A prospective, double-masked randomized clinical, sham-controlled study was carried out at the *Cai Ferate* Clinical Hospital in Iasi, Romania. The full study protocol complied in accordance with the 1964 Helsinki Declaration and its later amendments, and was approved by the local Ethics Committee of the *Cai Ferate* Clinical Hospital of Iasi, Romania (approval number: 54/29.07.2024) and retrospectively registered at ClinicalTrials.gov (NCT07067294) on 05.07.2025. No patients were involved in setting the research questions or the outcome measures, nor were they involved in developing plans for recruitment, design, or implementation of the study. No patients were asked to advise on interpretation or writing up of results. Prior to participation, all patients provided written informed consent.

Patients aged 60 years or older scheduled to undergo routine cataract surgery by phacoemulsification were screened for eligibility criteria and consecutively recruited between January and March 2025. Only one eye per patient was included in the study, specifically the eye scheduled for cataract surgery prior to enrollment. The study eye was selected without prior assessment of ocular surface status in order to reflect routine clinical practice and to evaluate the intervention under real-world conditions.

Exclusion criteria were: (i) previous ocular surgery in either eye, or any concomitant ocular pathology; (ii) current use of topical ocular medications (such as anti-inflammatory agents, artificial tears or immunomodulatory therapy) or device-based treatments in the last month; (iii) current or regular use of systemic medications associated with tear film alterations (including antihistamines, diuretics, antidepressants or hormone replacement therapy); (iv) use of contact lens in the last month; (v) autoimmune diseases (e.g., Sjögren’s syndrome); (vi) patients deemed temporarily unfit for surgery due to lack of cardiology clearance or laboratory findings suggestive of an acute systemic condition, requiring postponement and further medical evaluation; (vii) intraoperative or postoperative complications potentially affecting ocular surface integrity.

No significant changes to the study design, eligibility criteria, endpoints, or analysis plan were made after the study commenced. The extended protocol is provided in Supplementary File 1 and Supplementary Fig. 1. The corresponding CONSORT 2025 checklist is included in Supplementary File 2, and the CONSORT 2025 flow diagram^11^ is presented in Supplementary Fig. 2.

### Randomization and masking

Eligible participants were randomly assigned in a 1:1 ratio to each of the active low-level light treatment group or the sham-control group. Randomization was performed by an independent researcher/statistician who was not involved in the study using a computerized allocation sequence (http://www.sealedenvelope.com). Allocation concealment was ensured through sequentially numbered, opaque, sealed envelopes. Both patients and investigators responsible for clinical evaluations remained unaware of group assignment throughout the study period.

### Ocular surface workup

Before surgery, the ocular surface status of each participant was evaluated using a combined assessment of patient-reported symptoms and objective tear film tests. Clinical symptom severity was quantified using the Ocular Surface Disease Index questionnaire (OSDI). Tear film stability was evaluated by measuring tear break-up time (TBUT), while basal and reflex tear secretion were assessed using the Schirmer I test. Based on these parameters, diagnostic thresholds were applied in accordance with the Tear Film & Ocular Surface Society Dry Eye Workshop II (TFOS DEWS II) and Dry Eye Workshop III (TFOS DEWS III) recommendations^12,13^, and patients were categorized into 3 groups: (i) *normal* (OSDI score < 13, TBUT > 10 s, and Schirmer I ≥ 10 mm); (ii) *preclinica*l (OSDI score < 13 plus at least one between TBUT < 10 s or Schirmer I < 10 mm), and (iii) *DED* (OSDI score ≥ 13 plus at least one between TBUT < 10 s or Schirmer I < 10 mm).

### Treatment protocol

Participants allocated to the treated group underwent periocular LLLT using the Eye-light^®^ device (Espansione Marketing S.p.A., Bologna, Italy), which emits red light of 633 ± 10 nm wavelength. Two therapy sessions were administered for 15 min each, 7 days prior to cataract surgery and 7 days after surgery.

The control group received a sham procedure using the same device set to demo mode, delivering less than 30% of the therapeutic light intensity. This approach ensured patient masking while avoiding biologically meaningful light exposure. The trial ended when all randomized participants finished the course of the allocated treatment.

### Surgery and postoperative medical regimen

All cataract procedures were performed by a single experienced surgeon employing a standardized micro-incisional phacoemulsification technique (DCB). The study included one eye per patient, specifically the eye scheduled for cataract surgery prior to enrollment. Patients were recruited consecutively based on their surgical indication. The study eye was selected without a prior assessment of ocular surface status to better reflect routine clinical practice and to evaluate the intervention’s effectiveness under real-world conditions. Eyes with any intraoperative or postoperative aggravations were excluded from further analysis (CONSORT 2025 flow diagram – Supplementary Fig. 2). All patients followed an equivalent postoperative treatment protocol. This included a fixed combination of topical dexamethasone and netilmicin administered for 1 week, followed by a gradual taper of dexamethasone monotherapy over the subsequent 2 weeks. In addition, topical ketorolac was prescribed 3 times daily for 1 month. Preservative-free artificial tears containing cross-linked hyaluronic acid, trehalose, and sterilamine encapsulated in liposomes were administered 3 times daily for 3 months after the surgery.

### Outcome measures and follow-up schedule

The primary outcome of the present trial was to evaluate the effect of perioperatively administered LLLT on the Ocular Surface Disease Index (OSDI, 12 items questionnaire). The other study outcomes were structured into distinct categories to ensure methodological clarity. Secondary clinical endpoints comprised tear film breakup time (TBUT), corneal fluorescein staining, the Schirmer I test, and tear osmolarity (TearLab™ system), while exploratory endpoints included inflammatory and reparative biomarkers assessed in the tear film To further investigate these relationships, post hoc predictive analyses were performed to identify associations between baseline profiles and clinical responses.

Ocular surface evaluations (including the assessment of primary and secondary outcomes), as well as tear sampling, were performed at predefined time points: baseline, 1 week prior to surgery before the first treatment/sham session (T0), and 1 month following cataract surgery (T1). All assessments were carried out under the same environmental conditions by the same masked examiner (MMZT) to ensure methodological consistency and minimize bias. The study timeline is summarized in Supplementary Fig. 1.

To evaluate the efficacy of the intervention at T1, clinical improvement was predefined as a down-staging of disease severity: specifically, a transition from DED to preclinical or normal status, or from a preclinical condition to normal status.

During the treatment and follow-up period, safety and tolerability were systematically monitored at predefined time points using both subjective and objective assessments. Patients were actively queried regarding ocular symptoms (burning sensation, discomfort, pruritus, photophobia, tearing, and foreign body sensation), graded on a standardized 5-point scale (0–4) before, during, and immediately after each LLLT or sham session. Objective safety evaluations included intraocular pressure and visual acuity measurements performed at baseline, 1 week, and 1 month postoperatively, as well as slit-lamp assessment of conjunctival hyperaemia (Efron scale) and periocular erythema (Clinician’s Erythema Assessment scale) before and immediately after each session. Overall, no treatment-related adverse events or clinically relevant symptoms were reported in either group, and the intervention was well tolerated without interfering with the standard postoperative course.

### Tear sample processing

Tear samples were collected using Schirmer type I strips (Haag-Streit, UK) without topical anesthesia, following our previously reported protocol^14^. The strips were placed in the lower conjunctival fornix for up to 5 min, then individually stored in sterile microtubes at − 80 °C until further analysis in August 2025.

To ensure methodological consistency and reduce inter-assay variability, all tear samples were processed and analyzed together after completion of collection. More precisely, protein extraction was performed as follows: the strips were thawed and incubated in 400 µL of 1.5 M Tris–HCl buffer (pH 8.8) containing protease inhibitors for 3 h, followed by centrifugation at 16,000× g for 15 min (4 °C). Total protein content was determined by Nanodrop (Thermo Fisher Scientific, Waltham, USA), and tear cytokines and chemokines were quantified using a multiplex bead-based immunoassay (R&D Systems) on a Luminex FlexMap3D platform.

### Tear biomarker analysis

Tear biomarkers were quantified using a customized Luminex multiplex assay enabling the simultaneous measurement of growth differentiation factor 15 (GDF-15), β-nerve growth factor (β-NGF), vascular endothelial growth factor A (VEGF-A), platelet-derived growth factors (PDGF-AB and PDGF-CC), osteopontin (OPN), osteoprotegerin (OPG), and tumor necrosis factor alpha (TNF-α) in accordance with the manufacturer’s recommendations. Briefly, thawed tear samples were diluted 1:2 using the supplied sample diluent. Lyophilized analyte standards were reconstituted with calibrator diluent and combined to generate the highest concentration standard, followed by a three-fold serial dilution to obtain 6 standard points. The upper standard concentrations were 4,060 pg/mL for GDF-15, 940 pg/mL for β-NGF, 2,120 pg/mL for VEGFA, 6,140 pg/mL for PDGF-AB, 16,990 pg/mL for PDGF-CC, 258,220 pg/mL for OPN, 15,500 pg/mL for OPG, and 1,680 pg/mL for TNF-α. Assay procedures were otherwise performed as previously described^14^, with data acquisition carried out on a Luminex FlexMap3D system. Final biomarker concentrations were normalized to total tear protein content as determined by NanoDrop analysis.

### Statistical analysis

Statistical analyses were conducted using GraphPad Prism version 5 (GraphPad Software, San Diego, CA, USA) and SPSS version 27 (IBM SPSS Statistics, Chicago, IL, USA). The sample size was determined a priori based on the primary outcome, defined as the change in Ocular Surface Disease Index (OSDI, 12 items questionnaire) score between the treatment and sham groups. The calculation used a two-sided test for comparing mean changes between groups, with a significance level (α) of 0.05 and a statistical power of 80%. A clinically meaningful difference of 8 points in OSDI score was assumed, consistent with previously reported minimal clinically important differences^12,15,16^. Assuming a standard deviation of 12 points and a pre–post correlation of 0.5, the required sample size was estimated at 36 patients per group (total *n* = 72). To account for potential dropouts and preserve statistical power, 98 participants were initially enrolled. After attrition due to loss to follow-up (*n* = 5) or discontinuation of the intervention (*n* = 4), 45 participants remained in the LLLT group and 44 in the sham group. One participant in the LLLT group withdrew consent for tear sample collection for inflammatory biomarker analysis. Consequently, 88 participants (88 eyes; 44 per group) were included in the final analysis to ensure consistency across all reported data.

Data are generally presented as box-and-whisker plots illustrating minimum and maximum values. Tear biomarker distributions were initially assessed for normality and homogeneity of variance using the Shapiro–Wilk test. One patient who withdrew consent for tear sampling was excluded from the biomarker analyses, as tear collection could not be performed. Variables that met the assumptions of normality were further analyzed using two-way repeated-measures ANOVA, followed by Bonferroni post hoc correction for multiple comparisons. As most variables did not satisfy normality criteria, non-parametric statistical methods were applied: group comparisons were performed using the Kruskal–Wallis test with Dunn’s multiple-comparison post hoc analysis, while paired non-parametric data were evaluated using the Wilcoxon matched-pairs signed-rank test. Comparisons between two independent patient subgroups were conducted using the Mann–Whitney U test. Spearman’s rank correlation coefficients were calculated for non-parametric correlation analyses. Linear regression plots display the best-fit regression line together with the 95% confidence interval. Model fit was assessed using the coefficient of determination (R²), and statistical significance was determined by the F-test. For the analysis of tear film biomarkers, adjustments for multiple comparisons were performed to control the False Discovery Rate (FDR). Specifically, the Benjamini–Hochberg procedure was applied to account for the multiplicity of tests and reduce the likelihood of Type I errors; corresponding q-values were calculated in Python (version 3.12) for each biomarker across the study groups. Heat map visualizations were generated using OriginPro2024 software and were intended solely to provide a graphical overview of potential associations among tear biomarkers. Multivariate linear regression models incorporating clinical and paraclinical variables were developed to construct predictive mathematical models, whose ability to predict clinical improvement was evaluated using receiver operating characteristic (ROC) curve analysis in SPSS. To refine the predictive models and mitigate the risk of overfitting, we employed a LASSO (Least Absolute Shrinkage and Selection Operator) logistic regression analysis, which was implemented in Python (version 3.12) using the scikit-learn library. To ensure all biomarkers contributed equally to the regularization process regardless of their original units, all features were standardized using a StandardScaler prior to model fitting. The optimal regularization strength was determined through a stratified k-fold cross-validation procedure, using the SAGA solver and optimizing for the Area Under the Receiver Operating Characteristic curve (AUC). To maintain the reproducibility of our findings, a fixed random seed (random_state = 10) was applied. Statistical significance was defined as a p-value < 0.05.

## Results

A total of 315 patients were screened for eligibility within the clinical trial registered under NCT07067294. The study included one eye per patient, specifically the eye scheduled for cataract surgery prior to enrollment, and patients were recruited consecutively based on their surgical indication. Following the screening process, 98 eligible patients were recruited and randomized between January and March 2025: 50 were assigned to the low-level light therapy (LLLT) group and 48 to the sham treatment group (Supplementary Fig. 1). During the follow-up period, participants who were lost to follow-up (*n* = 5) or discontinued the intervention due to postoperative complications (*n* = 4) were excluded. Additionally, one patient in the LLLT group withdrew consent for tear sampling and was subsequently excluded from the biomarker evaluation. Consequently, the final analysis comprised 88 patients (88 eyes, 44 per group) who provided complete clinical and paraclinical data. The detailed flow of participants, including specific reasons for exclusion during the initial screening, is provided in the CONSORT flow diagram (Supplementary Fig. 2). Baseline demographic and clinical characteristics were balanced, with no statistically significant differences observed between the two study groups (Supplementary Table 1). The overall 88 patient cohort had a mean age of 73.8 ± 8 years and included 45 males and 43 females. In the LLLT group, the mean age was 73.4 ± 9 years, with 21 females and 23 males, and 21 right eyes and 23 left eyes included. In the sham group, the mean age was 74.2 ± 6.9 years, with an equal gender distribution (22 females and 22 males) and an equal number of right and left eyes (22 each).

Tear biomarkers and ocular surface examination (including OSDI 12 items questionnaire as the primary outcome measure, and the Schirmer’s test, tear breakup time (TBUT), corneal staining, and tear osmolarity as secondary outcomes) were assessed both at baseline (T0, Supplementary Table 1) and at 30 days post-cataract surgery (T1; Supplementary Table 2).

### Ocular surface changes in the LLLT and sham groups

OSDI, the primary outcome of the study, showed a significant decrease in the LLLT group at T1 (30 days post-cataract surgery), from a median value of 24 [CI: 12.25–38.75] to 11 [3.25–24.25] (*p* < 0.0001), while an increase was observed in the sham group, from 15 [CI: 12–27.25] to 24 [CI: 15–37.5] (*p* = 0.0152). When categorizing patients into preclinical and DED subgroups (as detailed in the Materials and Methods – Ocular Surface Workup section), improvement in OSDI in the LLLT group was observed only within the DED subgroup. The other ocular surface parameter that significantly improved was TBUT, which increased in both LLLT subgroups, from 5 [CI: 3.75–6.5] to 7.5 [CI: 5–12.5] seconds (*p* = 0.0139) in the preclinical subgroup and from 4 [CI: 3–6.5] to 7 [CI: 5–10] seconds (*p* = 0.002) in the DED subgroup (Supplementary Table 3).

In the sham group, tear osmolarity similarly increased in both subgroups, from 296 [CI: 293.3–303.8] to 306.5 [CI: 299.3–310] mOsm/L in the preclinical subgroup (*p* = 0.0011) and from 295 [288–300] to 302 [299–311] mOsm/L in the DED subgroup (*p* = 0.0005). Correction for multiple comparisons using the Benjamini–Hochberg method confirmed the significance of the changes listed above, while the other parameters did not show any significant changes over time within the indicated groups (Supplementary Table 3).

### Tear biomarkers changes in the LLLT and sham groups

**Fig. 1:**
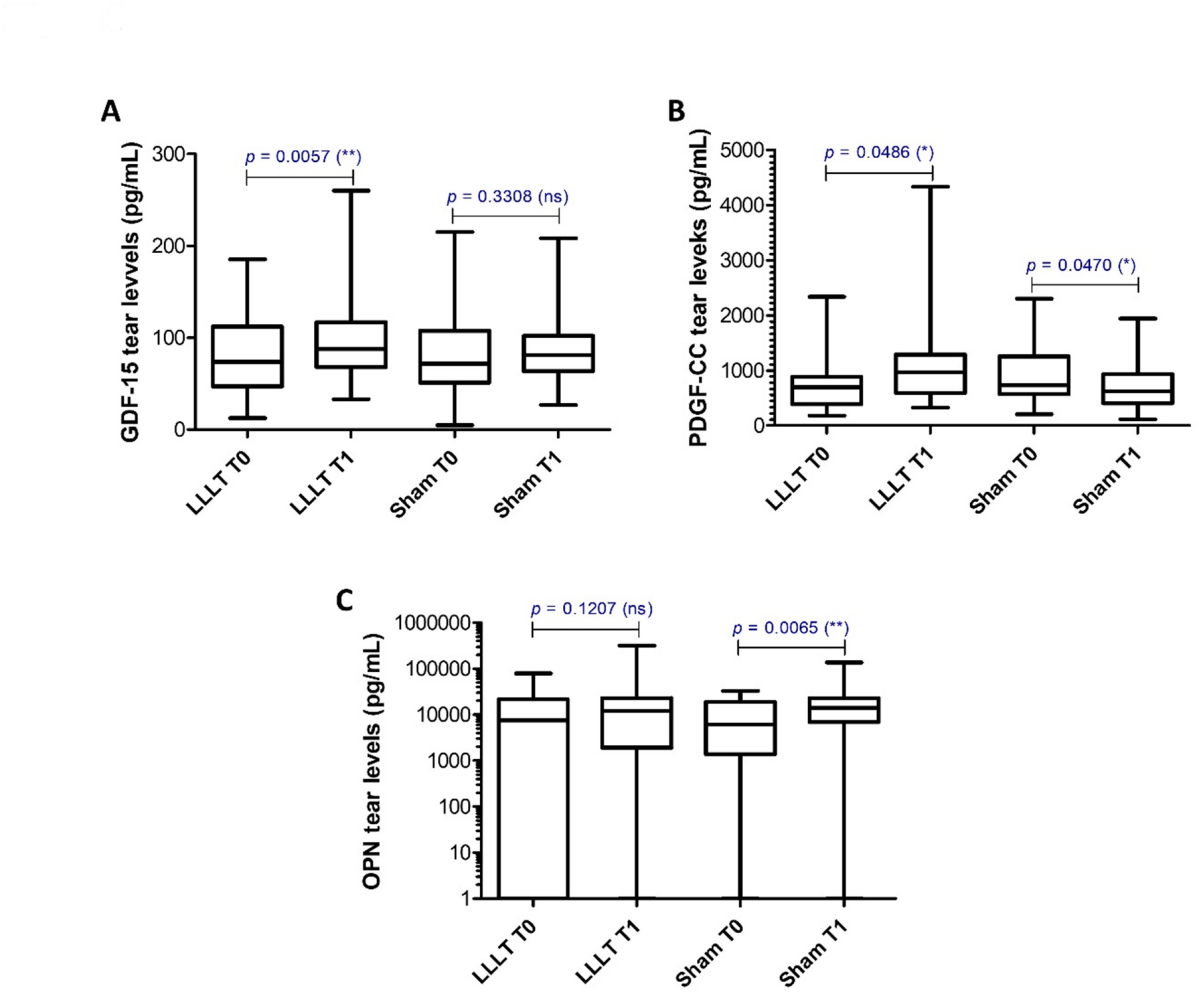
Tear levels of biomarkers showing significant changes over time in either LLLT or control (sham) subjects. Box and whisker plots indicating the minimum and maximum values for: (**A**) GDF-15; (**B**) PDGF-CC; (**C**) OPN (**p < 0.01, *p < 0.05, ns – not significant; two-tailed Wilcoxon matched pairs test).

The changes from T0 to T1 of the tear levels of investigated biomarkers in the two groups are shown in Figs. 1 (biomarker showing significant changes over time in either LLLT or control group) and 2 (biomarkers showing similar changes between LLLT and control groups). GDF-15 showed a significant increase in the LLLT group (from 73.62 [CI: 47.15–112] pg/mL at T0 to 87.8 [CI: 68.2–117] pg/mL at T1; *p* = 0.0057; Supplementary Table 2), while no significant changes were observed in the sham group (*p* = 0.3308) – Fig. 1A. Similarly, PDGF-CC showed an increase in the LLLT group (from 699.4 [CI: 390.3–879.8] pg/mL at T0 to 964.2 [CI: 593.6–1286] pg/mL at T1; *p* = 0.0486), while a significant reduction was observed in the sham group from 730.7 [CI: 573.6–1255] pg/mL at T0 to 623.7 [CI: 406.1–933.5] pg/mL at T1 (Fig. 1B). However, after correction for multiple comparisons using the Benjamini–Hochberg procedure, PDGF-CC changes were no longer statistically significant.

**Fig. 2:**
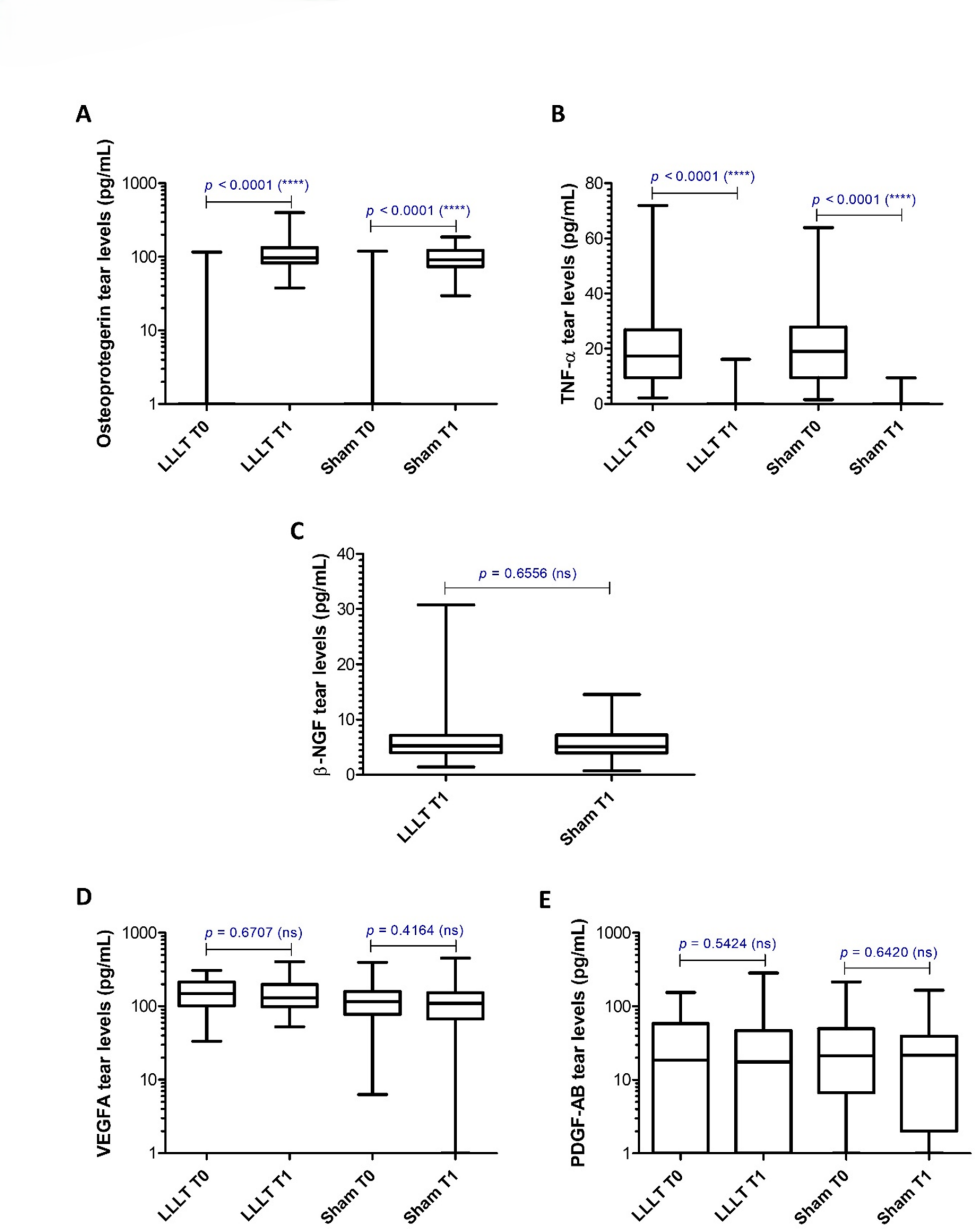
Tear levels of biomarkers showing similar dynamics over time in LLLT and control (sham) subjects. **(A-C)** Tear levels of biomarkers showing significant changes over time presented as box and whisker plots indicating the minimum and maximum values for: (**A**) Osteoprotegerin; (**B**) TNF-α; (**C**) β-NGF (****p < 0.0001, ns – not significant; two-tailed Wilcoxon matched pairs test). **(D-E)** Tear levels of biomarkers showing no significant change over time presented as box and whisker plots indicating the minimum and maximum values for: (**D**) VEGFA; **(E)** PDGF-AB (ns – not significant; two-tailed Wilcoxon matched pairs test).

Conversely, OPN exhibited a significant increase in the sham group, from 6052 [CI: 1389-18,985] pg/mL at T0 to 14,068 [CI: 6997-22,815] pg/mL at T1 (*p* = 0.0065; Fig. 1C), whereas no significant changes were observed in the LLLT group (*p* = 0.1207). OPG increased significantly in both groups to comparable levels, reaching 12.3 [CI: 1.93–23.17] ng/mL in the LLLT group and 14.07 [CI: 6.99–22.82] ng/mL in the sham group (both *p* < 0.0001; Fig. 2A). TNF-α decreased significantly in both groups (both *p* < 0.0001; Fig. 2B). β-NGF levels, which were undetectable at T0, became detectable at T1 in both the LLLT and sham groups with no significant differences between groups (Fig. 2C). The other growth factors analyzed (VEGFA and PDGF-AB) did not show significant changes over time (Fig. 2D-E).

**Fig. 3:**
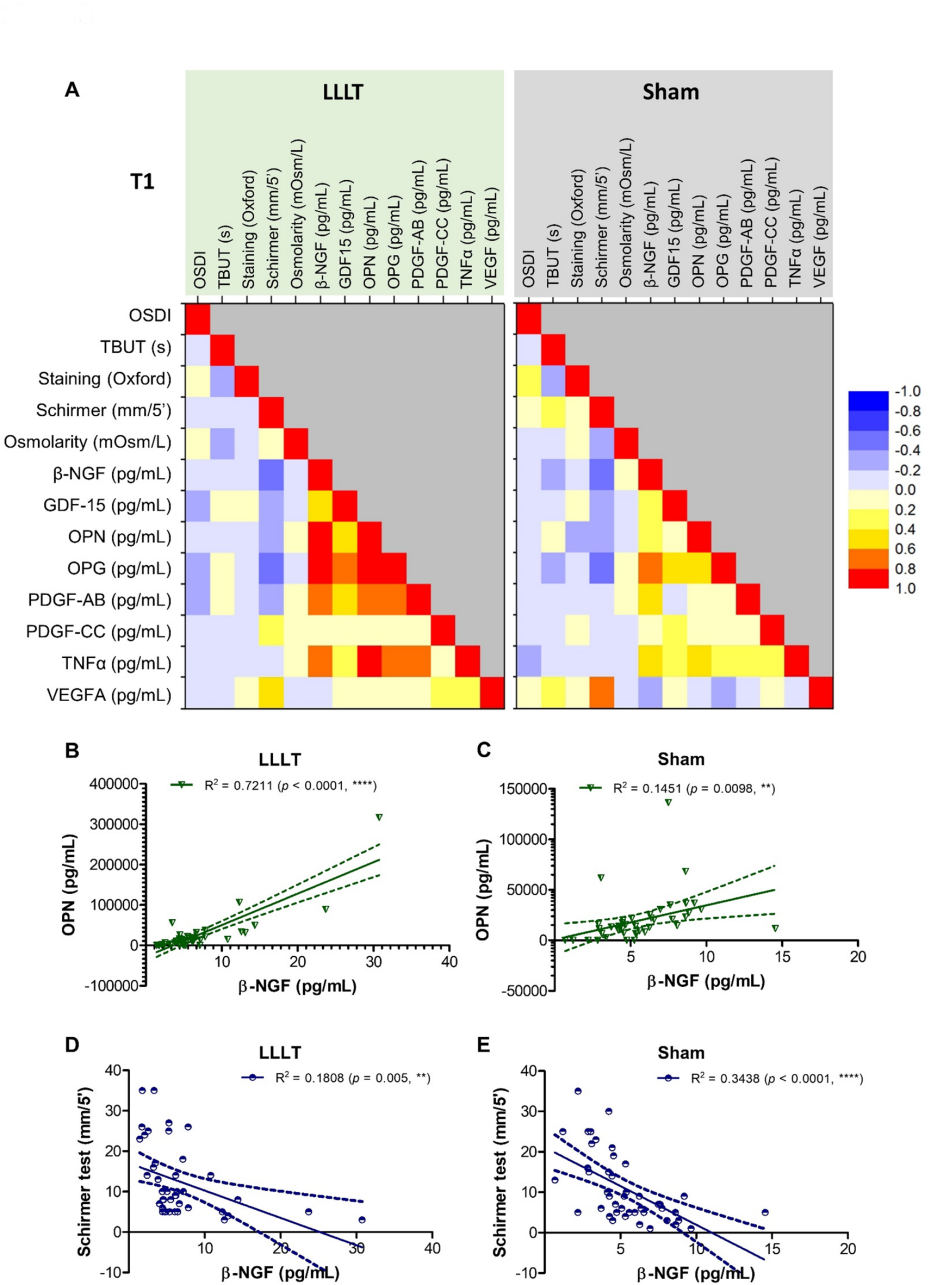
Regression statistic describing the association of distinct soluble biomarkers in tears in LLLT vs. controls (sham treatment). (**A**) Heat map of correlation coefficients (R) for LLLT (left) or sham cases (right) at T1 (after 30 days). Linear regression analysis for β-NGF and OPN in **(B)** LLLT and **(C)** sham cases, and for β-NGF and Schirmer test in **(D)** LLLT or **(E)** sham cases (****p < 0.0001, **p < 0.01; Spearman test).

When investigating the correlations between tear biomarkers at T1 in the two groups, a strong cluster of correlations was observed in the LLLT group between β-NGF and OPN, OPG, PDGF-AB, and TNF-α, while this cluster of associations was less pronounced in the control group (Fig. 3A-C). These tear biomarkers rather negatively correlated with OSDI and Schirmer test results (Fig. 3D-E). These findings suggest that the increase in β-NGF may contribute to counterbalancing the proinflammatory effects associated with OPN and TNF-α.

### Effects of LLLT on clinical outcomes

To evaluate the efficacy of the intervention at T1, clinical improvement was defined as a reduction in disease severity category, specifically a transition from DED to preclinical or normal status, or from preclinical to normal status. This categorical endpoint was intended to reflect clinically meaningful changes in disease status, consistent with established diagnostic frameworks for ocular surface disorders.

**Fig. 4:**
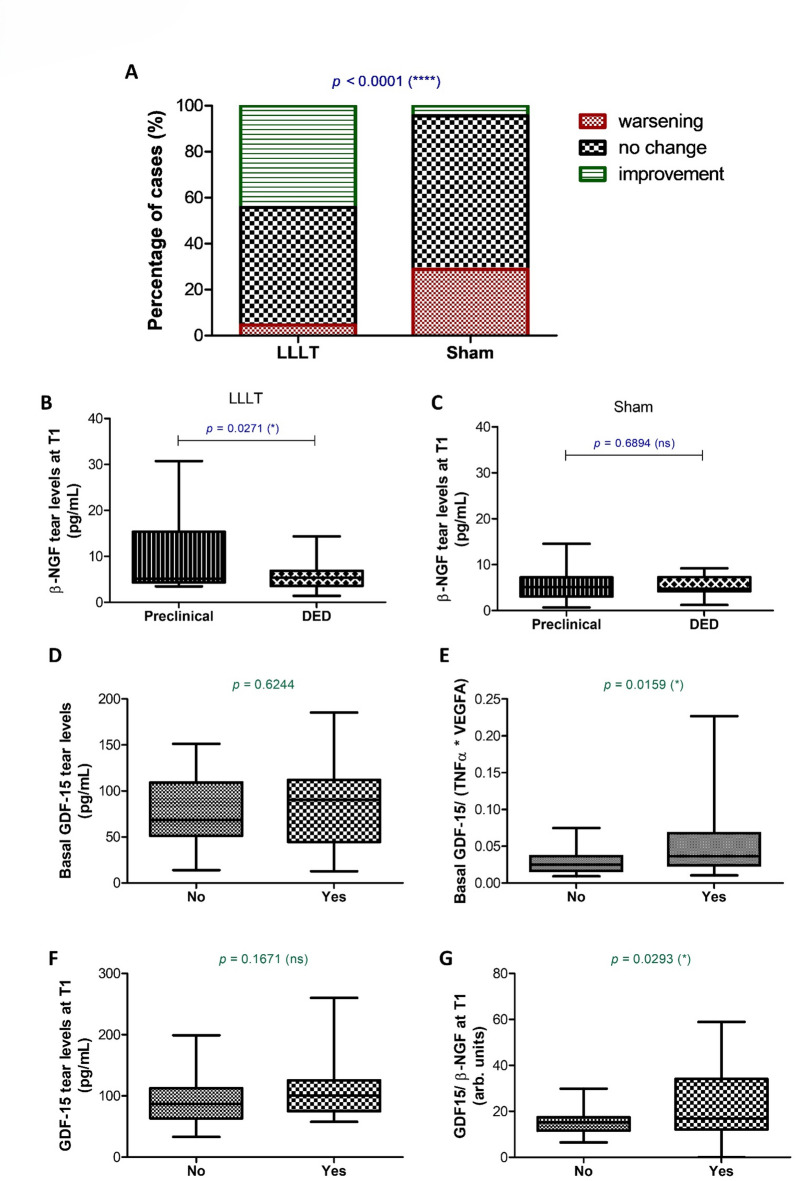
Clinical improvement of DED within the LLLT and sham groups 30 days post-cataract surgery. (**A**) Percentage of cases showing clinical improvement (****p < 0.0001; chi-squared test). **(B-C)** Changes in β-NGF tear levels in preclinical and clinical subgroups of patients for: (**B**) LLTT; **(C)** sham treatment (*p < 0.05, ns – not significant; Mann-Whitney U test). Box and whisker plots showing the minimum and maximum values for biomarkers in LLLT-treated eyes showing clinical improvement of DED: (**D**) basal GDF-15 levels; **(E)** basal GDF-15/(TNFα*VEGFA) ratio; **(F)** GDF-15 levels at T1 (one month after LLLT treatment); **(G)** GDF15/ β-NGF ratio at T1 (after one month of LLLT treatment) (*p < 0.05, ns – not significant; Mann-Whitney U test).

In the LLLT group, 44.2% of subjects showed improvement, compared with 4.4% in the sham group (*p* < 0.0001). In contrast, a higher rate of clinical worsening was observed in the sham group compared with the LLLT group (28.9% vs. 4.6%; Fig. 4A). Among the analyzed biomarkers, β-NGF showed a smaller increase in patients with initial DED (5.8 ± 0.55 pg/mL, *n* = 33) compared with preclinical subjects (10.1 ± 3.02 pg/mL, *n* = 11) within the LLLT group (*p* = 0.0271; Fig. 4B). In contrast, in the placebo group, the increase in β-NGF was comparable regardless of baseline DED status, with values of 5.52 ± 0.71 pg/mL in preclinical patients and 5.43 ± 0.42 pg/mL in DED patients (*p* = 0.6894; Fig. 4C). These findings suggest that LLLT, by inducing β-NGF increase, may confer greater benefit in preclinical patients than in those with baseline DED. The increases in tear levels of GDF-15 and PDGF-CC following LLLT were more visible in patients initially categorized as DED (Supplementary Tables 3 – upper panel: GDF-15 increased from 77.6 ± 7.2 to 95.5 ± 7.4 pg/mL (*p* = 0.0112), while PDGF-CC increased from 711.4 ± 76.6 to 1024 ± 130.8 pg/mL, *p* = 0.0473). In the sham group, tear levels of OPG and PDGF-CC showed a significant decrease over time in the preclinical subgroup, while the previously described increase in OPN was observed only in patients with baseline DED (Supplementary Tables 3 – bottom panel).

**Fig. 5:**
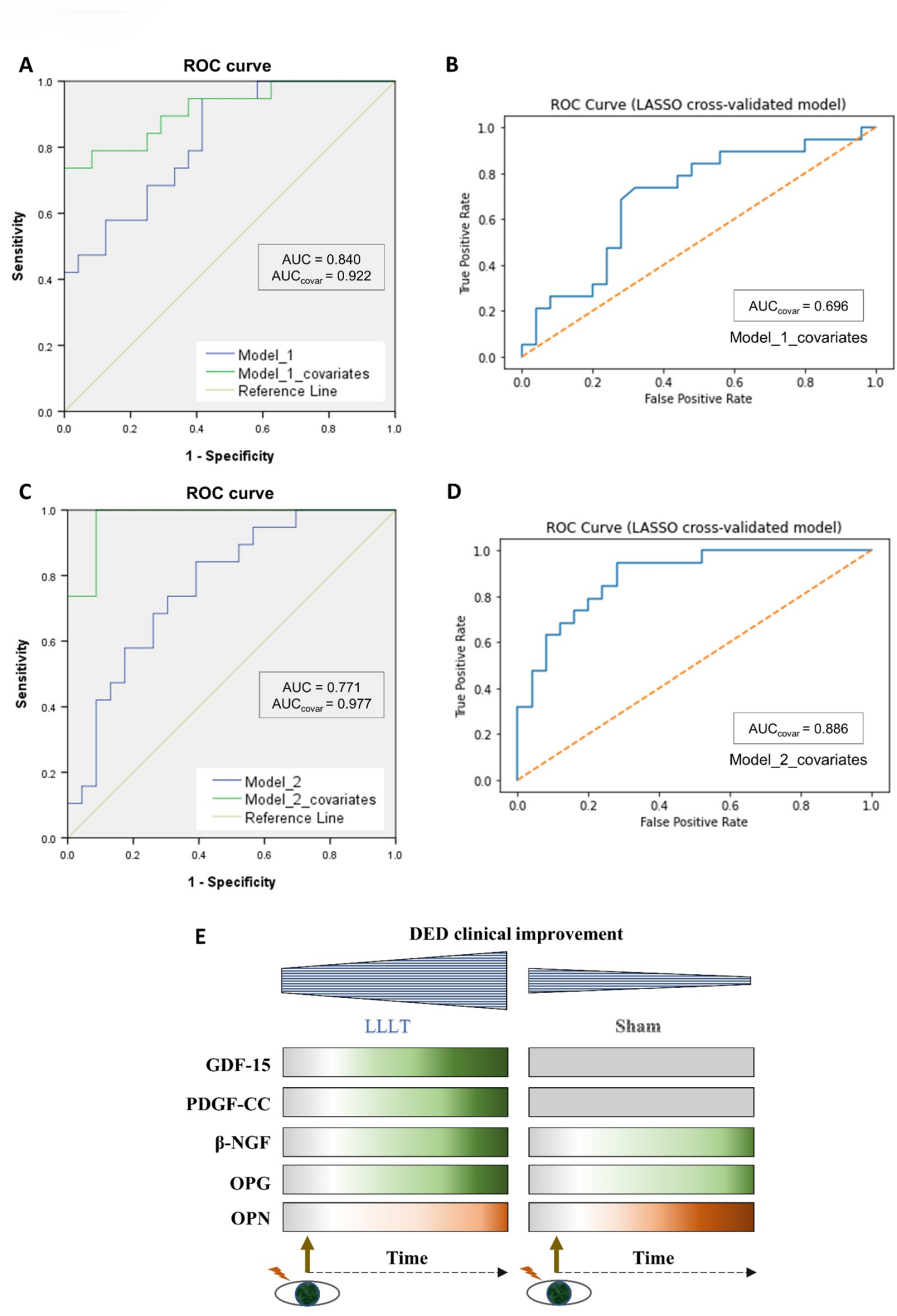
Mathematical models predicting clinical improvement in LLLT-treated patients. Representation of the generated ROC curves for: **(A)** Model_1 based on basal clinical and paraclinical parameters: basal osmolarity, GDF-15, OPN, TNF-α, and PDGF-CC; covariates include age, gender, and basal clinical status; **(B)** LASSO-refined Model_1; **(C)** Model_2 based on clinical and paraclinical parameters at T1 (30 days post-LLLT treatment): osmolarity, GDF-15, β-NGF, TNF-α, and VEGFA; covariates include age, gender, and clinical status; **(D)** LASSO-refined Model_2. **(E)** Schematic of clinical and biomarker responses to low-level light therapy (LLLT) versus sham treatment in cataract surgery. Biomarkers associated with tissue-repair (GDF-15, PDGF-CC, β-NGF, OPG) increased more with LLLT, while the proinflammatory marker OPN rose more with sham treatment, highlighting the importance of balancing proinflammatory and tissue-repair pathways for improved outcomes in early dry eye disease (DED).

In the LLLT group, patients who showed clinical improvement had higher baseline levels of GDF-15 and slightly lower levels of TNF-α and VEGFA (Fig. 5D, Supplementary Table 4). Accordingly, the composite ratio GDF-15/(TNF-α × VEGFA) may represent a novel biomarker for predicting the efficacy of LLLT (Fig. 5E). This ratio increased from 0.025 [CI: 0.016–0.036] in patients without improvement to 0.037 [CI: 0.024–0.068] in the improved group. At T1, the improved group exhibited higher levels of both GDF-15 and β-NGF (Fig. 5F, Supplementary Table 4). However, the balance between these biomarkers, expressed as the GDF-15/β-NGF ratio, remained higher in patients with clinical improvement, suggesting a key role for GDF-15 and for the relative balance between these mediators. Specifically, patients without improvement had a GDF-15/β-NGF ratio of 15.22 [CI: 11.55–17.46], whereas the improved group showed a higher ratio of 16.98 [CI: 12.07–34.11] – Fig. 5G. Thus, these two novel ratios may represent potential tear biomarkers for predicting the efficacy of LLLT in reducing DED following cataract surgery. In subjects who did not achieve overall clinical improvement (as defined above), both OSDI and TBUT still showed significant improvement after Benjamini–Hochberg correction, although the magnitude of change was not sufficient to reach the normal range. Importantly, clinical improvement was associated with a significant decrease in baseline tear osmolarity, from 309 [CI: 293–318] mOsm/L at T0 to 299 [CI: 286–309] mOsm/L at T1 (*p* = 0.0055; Supplementary Table 4).

### Integration of tear biomarkers into mathematical models for predicting LLLT efficacy

Linear regression models incorporating baseline tear osmolarity, GDF-15, OPN, TNF-α, and PDGF-CC achieved excellent predictive performance, with an area under the curve (AUC) of 0.840 (Fig. 5A, Supplementary Table 5). Among these biomarkers, baseline TNF-α proved as a negative independent predictor while basal tear osmolarity as a positive predictor for clinical improvement (Supplementary Table 6). After adjustment for potential covariates, including age, sex, and baseline clinical status, the model demonstrated excellent predictive capacity, reaching an AUC of 0.922. To ensure the robustness of our findings and reduce the risk of overfitting, Least Absolute Shrinkage and Selection Operator (LASSO) regression was applied for variable selection and model regularization. Although the initial unadjusted model achieved an AUC of 0.922, the LASSO approach provided a more conservative and generalizable estimate of predictive performance. The main predictors identified through this method were consistent with those included in the initial model, yielding a refined model with an AUC of 0.696 (Fig. 5B). This performance reflects a balanced trade-off between model complexity and generalizability, providing a more realistic estimate of the biomarkers’ predictive value in clinical practice.

To further identify which parameters measured at T1 best predicted improvement and thus contributed to understanding the immune and molecular mechanisms underlying the effects of LLLT on clinical outcomes, additional models including final tear osmolarity, GDF-15, β-NGF, TNF-α, and VEGFA were developed (Fig. 5C, Supplementary Table 5). This model achieved good predictive performance (AUC = 0.771), which increased markedly after adjustment for covariates such as age, sex, and clinical status, resulting in an AUC of 0.977. Among all these biomarkers, β-NGF at T1 emerged as a positive indicator of clinical improvement following LLLT (Supplementary Table 7). To reduce the risk of overfitting and improve model generalizability, LASSO regression was subsequently applied. This approach yielded a model with an AUC of 0.886 and retained all predictors except GDF-15 (Fig. 5D), suggesting that the remaining variables contributed more robustly to outcome prediction at 30 days post-cataract surgery.

Thus, we conclude that LLLT may be associated with increases in both GDF-15 and β-NGF tear levels, but with a more pronounced increase in GDF-15. In preclinical subjects, the therapy primarily elevates β-NGF, whereas in subjects with baseline DED, more prominent changes are observed in GDF-15 and PDGF-CC. The GDF-15/β-NGF ratio therefore appears to be relevant in characterizing treatment response.

### Safety and tolerability

Safety and adverse events were prospectively and systematically assessed at predefined time points, using both subjective and objective measures. Patients were actively queried regarding the presence of possible ocular symptoms, including burning sensation, discomfort, pruritus, photophobia, tearing, and foreign body sensation, which were graded on a standardized 5-point scale (0–4) and the answers recorded before, during, and immediately after each LLLT or sham session. Objective safety evaluations included intraocular pressure and visual acuity measurements performed at baseline, 1 week, and 1 month after cataract surgery. In addition, conjunctival hyperaemia (Efron scale, 0–4) and periocular erythema (Clinician’s Erythema Assessment - CEA scale, 0–4) were assessed before and immediately after each LLLT or sham procedure, while skin pigmentation was monitored using a standardized 5-point clinical grading scale. A mild and transient (grade 1) conjunctival and periocular erythema was occasionally observed immediately after LLLT treatment in 8 subjects, but resolved spontaneously within minutes. No cases of hyperpigmentation or persistent pigmentary changes were detected during treatment or follow-up. Overall, no treatment-related adverse events or clinically relevant symptoms were reported in either group, and the procedure was well tolerated without interfering with the standard postoperative course.

## Discussions

The present study reported several molecular changes following LLLT that provide novel insights into the mechanisms stimulated by this technology for tissue repair, neurotrophic support, and inflammation regulation at the ocular surface. Furthermore, the study demonstrated that LLLT-treated patients showed a higher rate of clinical improvement one month after cataract surgery compared with sham patients. However, these findings should be interpreted with caution, as all patients received a postoperative treatment regimen that may have influenced ocular surface status and tear biomarker profiles.

The comprehensive assessment of ocular surface health revealed significant improvement following LLLT in TBUT in preclinical cases and in both TBUT and OSDI in subjects with baseline DED. Conversely, in the sham group, tear osmolarity significantly increased in both subgroups, indicative of DED progression.

Regarding tear biomarkers, patients treated with LLLT exhibited a significant increase in GDF-15 and PDGF-CC tear levels, particularly among those with DED at baseline, whereas increases in β-NGF were more prominent in patients with preclinical conditions. While these patterns may suggest distinct and potentially complementary roles for these molecules in tissue recovery following surgical trauma, a contributory effect of postoperative co-interventions cannot be excluded.

GDF-15, a stress-induced cytokine from the transforming growth factor-β superfamily, is upregulated in response to tissue injury and inflammation and has been implicated in pro-regenerative processes and modulation of wound healing^17,18^. The more pronounced increase in GDF-15 observed in patients with DED treated with LLLT may reflect enhanced activation of intrinsic anti-inflammatory and tissue modulation pathways, facilitating controlled repair and resolution of inflammation. GDF-15 has been shown to attenuate epithelial–mesenchymal transition and reduce fibrosis in lens models, suggesting a potential role in modulating post-surgical wound responses in the eye^17^. Its regulatory function includes modulation of apoptosis, cell proliferation, and inflammatory signaling, which may help shifting the local microenvironment toward a reparative state^18^.

β-NGF is a neurotrophin essential for neuroprotection, corneal epithelial integrity, and tissue remodeling. Although its expression in tear fluid and ocular tissues is less well characterized than in neural tissues, it has been associated with regenerative processes, modulation of inflammation, and neurotrophic support in ocular disease contexts^19–22^. The preferential increase in β-NGF among patients with preclinical conditions may reflect early modulation of neural and trophic signaling in response to LLLT, which could be relevant for the maintenance of ocular surface integrity and potentially preventing progression to DED. β-NGF may act synergistically with GDF-15, balancing pro-reparative and anti-inflammatory signals, thereby enhancing epithelial recovery, nerve regeneration, and tear film stability. It also modulates local immune responses by promoting epithelial survival and reducing apoptosis, contributing to improved ocular comfort and functional outcomes following surgery^19–22^.

Comparison of PDGF isoforms revealed a selective increase in PDGF-CC following LLLT, whereas no significant changes were observed PDGF-AB. PDGFs are key drivers of cell proliferation, migration, and angiogenesis during wound healing^23^. While all isoforms contribute to early repair, PDGF-CC preferentially binds PDGFR-αα and PDGFR-αβ, stimulating fibroblast and pericyte proliferation and migration, mechanisms which are essential for extracellular matrix (ECM) remodeling and vascular stabilization^24,25^. PDGF-CC is also involved in repairing corneal tissue and neural regeneration in the eye, supporting its relevance in tear film dynamics^26^. Its presence in tears suggests a role in the ocular response to injury or disease, including DED or graft-versus-host disease^27^. In contrast, PDGF-AB plays a key role in corneal stroma and epithelium repair, and is commonly present as a heterodimer in platelet-rich plasma eye drops^28,29^. Additionally, PDGF-CC is secreted as an inactive precursor requiring proteolytic activation, whereas PDGF-AB, although mitogenic, is more strongly associated with early inflammatory cell recruitment rather than later ECM maturation^28^. Animal studies have shown that PDGF-CC promotes re-epithelialization and granulation tissue formation, further supporting its role in tissue regeneration^30,31^. The selective increase in PDGF-CC observed in LLLT-treated eyes may reflect the activation of reparative pathways promoting organized tissue reconstruction and epithelial restoration following cataract surgery, whereas PDGF-AB dynamics appear less critical in later-stage matrix stabilization. In the ocular surface context, where a fine balance between transparency, epithelial integrity, and immune surveillance is required, these distinctions are particularly meaningful. LLLT has been proposed to modulate cellular bioenergetics and cytokine networks, enhancing reparative signaling while attenuating excessive inflammation^32^. The absence of significant changes in PDGF-AB suggests that LLLT may primarily influence on downstream processes such as ECM remodeling and cellular proliferation rather than early inflammatory recruitment, which may partly explain the improved biomarker and clinical profiles observed compared with placebo.

In contrast, the sham group exhibited tear biomarker dynamics consistent with persistent, uncontrolled inflammation. Specifically, OPN increased in patients with baseline DED, reflecting ongoing immune activation and fibrogenic signaling, whereas OPG decreased in patients with preclinical conditions, suggesting diminished anti-inflammatory regulation.

OPN is a multifunctional matricellular protein that is upregulated in the setting of tissue injury and chronic inflammation^33,34^. It acts both as a cytokine and as an extracellular matrix component, contributing to immune cell recruitment and activation, fibroblast migration, and extracellular matrix deposition in various tissues. Elevated OPN levels have been linked to persistent inflammatory and fibrogenic responses in a range of ocular and systemic conditions, and may therefore reflect a sustained injury signal rather than resolution of inflammation^35,36^. Its association with angiogenic and fibrogenic mediators in proliferative ocular disorders, further supports this interpretation^39^. By contrast, OPG, traditionally recognized for its role in bone homeostasis as a decoy receptor for Receptor Activator of Nuclear factor Kappa-B Ligand (RANKL), also intersects with inflammatory and vascular pathways^37–39^. Within inflammatory signaling, the RANK/RANKL/OPG axis play a significant role in immune regulation^40^. Some studies suggest that, through interactions with RANKL, as well as TNF-related apoptosis inducing ligand (TRAIL), OPG may promote angiogenesis^41,42^. In proliferative diabetic retinopathy, OPG expression correlates with angiogenic and inflammatory factors such as VEGF and MCP-1, and has been shown to potentiate endothelial cell migration via ERK1/2 and Akt signaling pathways, suggesting involvement in both inflammation and angiogenic regulation^43^. Overall, OPG may serve a dual role: promoting cell survival through binding and neutralizing TRAIL, while limiting cell proliferation and inflammation via inhibition of the RANKL pathway^37,44^. Although its role at the ocular surface remains incompletely understood, reduced OPG levels in the sham preclinical subgroup may point towards a lower capacity to counterbalance inflammatory stimuli.

An important consideration when interpreting the observed biomarker dynamics is the standardized postoperative treatment protocol applied uniformly to both the LLLT and sham groups. All patients followed the same postoperative treatment protocol, which included a short course of combined topical dexamethasone and netilmicin during the first postoperative week, followed by a stepwise tapering of dexamethasone over the subsequent two weeks, alongside non-steroidal anti-inflammatory drops administered three times daily for one month. In addition, preservative-free artificial tears (containing cross-linked hyaluronic acid, trehalose, and liposome-encapsulated sterilamine) were used for up to three months post-surgery. Given that these co-interventions are known to augment autophagy to modulate ocular surface inflammation and tear film composition^45,46^, they likely contributed to the overall clinical improvement observed in both cohorts. However, because high-dose corticosteroid exposure was limited to the early postoperative period (first 7 days), whereas biomarker assessment was performed at the 30 days after cataract surgery, the remaining between-group differences in biomarker trajectories likely reflect the additive effect of LLLT rather than the baseline regimen alone. While these findings suggest an additional modulatory role of photobiomodulation on inflammatory and reparative pathways, causal attribution solely to LLLT should be interpreted with caution since an additive or synergistic effect between the postoperative regimen and LLLT cannot be excluded. Although not a primary outcome, some patients experienced improvements in periocular skin quality and reduced edema, without any adverse effects such as periocular skin hyperpigmentation, further supporting the safety profile of the intervention.

Overall, LLLT therapy was associated with elevated tear levels of both GDF-15 and β-NGF; however, the increase in GDF-15 was more pronounced, suggesting that the relative balance between these two biomarkers may also be important in predicting LLLT efficacy. Thus, it might be possible that those two biomarkers to contribute in mitigating the pro-inflammatory activity associated with OPN and TNF-α. Exploratory analysis of baseline biomarkers combined into composite ratios suggested that the GDF-15 / (TNF-α × VEGFA) index could be associated with LLLT efficacy and with the progression of DED, although these findings require further validation. Additionally, LLLT appeared to provide greater benefit in patients with established DED at baseline compared with those presenting with preclinical symptoms at baseline.

This study has several limitations. First, the sample size (two groups of 44 participants each) is sufficient to detect moderate to large differences in the primary tear biomarkers, but smaller effects may not be detected, limiting the power to identify subtle changes. Second, while tear biomarkers can exhibit some biological variability between individuals and across collection times, this variability is expected to be moderate and was partially controlled through standardized collection procedures. The study was conducted at a single center, which may limit the generalizability of the findings, and although a sham group was used, placebo effects cannot be completely excluded. Finally, the follow-up period was relatively short, which may not capture long-term effects of LLLT on ocular biomarkers.

## Conclusions

Taken together, the observed differences in inflammatory regulators (OPN, OPG) and reparative growth factors (GDF-15, β-NGF, PDGF-CC) between LLLT and sham groups suggest distinct biological responses. In LLLT-treated eyes, the concurrent modulation of growth factors and neurotrophic signaling may reflect activation of reparative pathways and restoration of ocular surface homeostasis, whereas a more persistent pro-inflammatory signaling was observed in the sham group.

The combined regulation of GDF-15 and β-NGF may be particularly relevant: β-NGF is associated with epithelial and neurotrophic support, while GDF-15 is considered a stress-responsive mediator involved in regulated tissue repair processes. PDGF-CC may complement these effects by contributing to ECM remodeling and epithelial proliferation.

Overall, these findings suggest that LLLT may modulate a network of tear biomarkers related to inflammation, tissue remodeling, and neurotrophic support, with GDF-15, β-NGF, and PDGF-CC emerging as potentially relevant components, as summarized in Fig. 5E. Exploratory analyses further suggest that LLLT may be beneficial in patients with higher baseline GDF-15 and lower VEGFA levels, as reflected by the composite biomarker ratio GDF-15 / (TNF-α × VEGFA); however, this finding should be interpreted cautiously given the post hoc nature of the analysis. This coordinated response likely underlies the improved clinical outcomes observed in LLLT-treated patients, supporting its potential as a post-surgical therapeutic intervention^47,48^. Importantly, changes in the dynamics of these biomarkers correlated with improvements in clinical parameters such as OSDI score, TBUT, and Schirmer’s test. Future studies are warranted to clarify the mechanistic interactions of these biomarkers at the cellular level and to evaluate their potential predictive value for post-surgical recovery.

## Supplementary Information

Below is the link to the electronic supplementary material.

Supplementary Material 1

Supplementary Material 2

Supplementary Material 3

Supplementary Material 4

## Data Availability

The relevant data for this study are included within the manuscript files. The datasets generated and analyzed during the current study are available from the corresponding author upon reasonable request.
