# Supplementary material for: Tear biomarker changes and ocular surface recovery with low-level light therapy after cataract surgery: a double-masked randomized controlled clinical trial
Source: Sci Rep. 2026 May 20;16:22977. doi: 10.1038/s41598-026-53521-4 (PMC13391487; doi:10.1038/s41598-026-53521-4)
Supplement: Supplementary file 1 — Supplementary Material 1 [file 41598_2026_53521_MOESM1_ESM.docx]

**Supplementary Table 1. Baseline characteristics of patients undergoing cataract surgery who received low-level light therapy (LLLT) or sham treatment.**

| **Baseline parameters** | **LLLT** | | **Sham** | | **p-value** | **q-value** | |
| --- | --- | --- | --- | --- | --- | --- | --- |
|  | **Median [CI]** | **mean ± s.e.m.** | **Median [CI]** | **mean ± s.e.m.** |  |  |  |
| Age (years) | 75 [67.25-79.5] | 73.36 ± 9.004 | 75 [70-77.75] | 74.16 ± 6.931 | 0.6436**^‡^** | 0.8275 |  |
| Gender | F = 21  M = 23 | | F = 22  M = 22 | | 1.000* | 1.000 | |
| Eye selected for surgery | LE =23  RE = 21 | | LE =22  RE = 22 | | 1.000* | 1.000 | |
| Ocular surface status | Preclinical = 11  DED = 33 | | Preclinical = 20  DED = 24 | | 0.0734* | 0.1476 | |
| OSDI | 24 [12.3-38.8] | 26.5 ± 15.6 | 15 [12-27.3] | 20.8 ± 12.7 | 0.1154**^‡^** | 0.3462 |  |
| TBUT (s) | 4 [3-7.5] | 5.8 ± 4.0 | 5.5 [4.3-10] | 7.1 ± 3.7 | 0.1107**^‡^** | 0.3303 |  |
| Corneal Staining (Oxford) | 0 [0-0] | 0.227 ± 0.605 | 0 [0-0.75] | 0.284 ± 0.522 | 0.3296**^‡^** | 0.5933 |  |
| Schirmer test (mm/5') | 8 [4.3-13] | 10.4 ± 8.1 | 7 [5-13.8] | 9.3 ± 7.6 | 0.5383**^‡^** | 0.8075 |  |
| Tear osmolarity (mOsm/L) | 299 [290.5-309.8] | 300.1 ± 13.9 | 296 [290.5-301] | 296.7 ± 15.7 | 0.2729**^‡^** | 0.5933 |  |

**^‡^** Mann-Whitney test; *chi-squared test; OSDI, Ocular Surface Disease Index; TBUT, tear film break-up time; DED = dry eye disease; q-values represent p-values corrected for multiple comparisons using the Benjamini-Hochberg procedure.

**Supplementary Table 2. Overall dynamics of ocular biomarkers in patients undergoing cataract surgery who received low-level light therapy (LLLT) or sham treatment.**

| **Treatment** | **T0** | | **T1** | | **p-value^‡^** | **q-value** |
| --- | --- | --- | --- | --- | --- | --- |
|  | **Median [CI]** | **mean ± s.e.m.** | **Median [CI]** | **mean ± s.e.m.** |  |  |
| **LLLT** | | | | | | |
| GDF-15 (pg/mL) | 73.62 [47.15-112] | 79.91 ± 6.301 | 87.77 [68.23-116.9] | 98.66 ± 6.871 | **0.0057** | **0.0114** |
| OPN (pg/mL) | 7580 [0-21455] | 13138 ± 2554 | 12297 [1926-23172] | 25086 ± 7924 | 0.1207 | 0.1609 |
| OPG (pg/mL) | 0 [0-0] | 3.789 ± 2.779 | 96.63 [82.25-132.8] | 112.7 ± 9.769 | **<0.0001** | **0.0003** |
| PDGF-AB (pg/mL) | 18.54 [0-58.53] | 32.33 ± 6.191 | 17.49 [0-46.64] | 37.19 ± 9.17 | 0.5424 | 0.6199 |
| PDGF-CC (pg/mL) | 699.4 [390.3-879.8] | 814.8 ± 85.44 | 964.2 [593.6-1286] | 1043 ± 108.4 | **0.0486** | 0.0778 |
| TNF-α (pg/mL) | 17.34 [9.504-26.91] | 20.04 ± 2.265 | 0 [0-0.03813] | 0.7676 ± 0.4316 | **<0.0001** | **0.0003** |
| VEGFA (pg/mL) | 148.8 [101.2-214.9] | 158.6 ± 11.22 | 130.3 [98.75-197.5] | 160.6 ± 13.51 | 0.6707 | 0.6707 |
| β-NGF (pg/mL) | 0 [0-0] | 0 ± 0 | 5.26 [3.98-7.13] | 6.77 ± 0.86 | **<0.0001** | **0.0003** |
| **Sham** | | | | | | |
| GDF-15 (pg/mL) | 71.96 [51.13-107.5] | 84.05 ± 6.982 | 81.01 [63.55-102.2] | 87.2 ± 5.527 | 0.3308 | 0.4411 |
| OPN (pg/mL) | 6052 [1389-18985] | 10143 ± 1507 | 14068 [6997-22815] | 19376 ± 3446 | **0.0065** | **0.0130** |
| OPG (pg/mL) | 0 [0-0] | 2.875 ± 2.602 | 90.7 [73.55-122.2] | 96.17 ± 5.303 | **0.0001** | **0.0003** |
| PDGF-AB (pg/mL) | 21.31 [6.691-49.55] | 35.29 ± 6.487 | 21.51 [2.016-39.28] | 28.91 ± 4.721 | 0.642 | 0.6420 |
| PDGF-CC (pg/mL) | 730.7 [573.6-1255] | 909.4 ± 78.53 | 623.7 [406.1-933.5] | 724 ± 60.94 | **0.047** | 0.0752 |
| TNF-α (pg/mL) | 19.03 [9.558-27.91] | 20.84 ± 2.062 | 0 [0-0] | 0.5182 ± 0.2513 | **0.0001** | **0.0003** |
| VEGFA (pg/mL) | 115.8 [77.93-159.1] | 131.4 ± 12.54 | 109.8 [67.39-152.6] | 128.3 ± 13.58 | 0.4164 | 0.4759 |
| β-NGF (pg/mL) | 0 [0-0] | 0 ± 0 | 5.08 [3.93-7.23] | 5.47 ± 0.39 | **0.0001** | **0.0003** |

**^‡^** Wilcoxon matched-pairs signed-rank test; GDF-15, Growth differentiation factor 15; OPN, osteopontin; OPG, osteoprotegerin; PDGF, Platelet-Derived Growth Factor; TNF-α, Tumor Necrosis Factor alpha; VEGFA, Vascular Endothelial Growth Factor A; β-NGF, Nerve Growth Factor-beta; q-values represent p-values corrected for multiple comparisons using the Benjamini-Hochberg procedure.

**Supplementary Table 3. Dynamics of ocular parameters in patients undergoing cataract surgery who received low-level light therapy (LLLT) or sham treatment.**

| **Treatment** | **T0** | | **T1** | | **p-value^‡^** | **q-values** |
| --- | --- | --- | --- | --- | --- | --- |
|  | **Median [CI]** | **mean ± s.e.m.** | **Median [CI]** | **mean ± s.e.m.** |  |  |
| **LLLT** | | | | | | |
| **Preclinical** | | | | | | |
| OSDI | 9.5 [6-11] | 8.6 ± 0.896 | 3.5 [0-8] | 6 ± 2.963 | 0.0821 | 0.1916 |
| TBUT (s) | 5 [3.75-6.5] | 5.8 ± 1.153 | 7.5 [5-12.5] | 9.4 ± 1.91 | **0.0139** | **0.0487** |
| Corneal Staining (Oxford) | 0 [0-0] | 0 ± 0 | 0 [0-0] | 0.1 ± 0.1 | 1.000 | 1.0000 |
| Schirmer test (mm/5') | 6.5 [3-9.25] | 6.5 ± 1.025 | 5.5 [4.5-9.5] | 8.4 ± 2.418 | 0.5745 | 0.7312 |
| Tear osmolarity (mOsm/L) | 301 [288.8-310] | 299.1 ± 4.515 | 293 [279.8-300] | 290.2 ± 3.96 | 0.0578 | 0.1618 |
| conc. proteins (mg/dL) | 0.43 [0.36-0.56] | 0.47 ± 0.045 | 0.49 [0.24-0.60] | 0.43 ± 0.06 | 0.4922 | 0.6891 |
| GDF-15 (pg/mL) | 71.92 [61.36-134.9] | 87.56 ± 13.33 | 101.3 [71.5-137.5] | 110.7 ± 15.76 | 0.2051 | 0.3190 |
| OPN (pg/mL) | 7837 [196.7-16469] | 14507 ± 7500 | 23402 [6081-64506] | 56756 ± 30218 | 0.1055 | 0.2110 |
| OPG (pg/mL) | 0 [0-0] | 2.985 ± 2.985 | 126.1 [86.64-190.5] | 152.8 ± 31.16 | **0.002** | **0.0140** |
| PDGF-AB (pg/mL) | 22.3 [0-93.67] | 44.89 ± 19.28 | 27.65 [5.263-86.02] | 60.3 ± 27.73 | 0.8203 | 0.8834 |
| PDGF-CC (pg/mL) | 760.8 [610.3-1500] | 1056 ± 214.3 | 1017 [622.9-1455] | 1068 ± 158.7 | 0.625 | 0.7292 |
| TNF-α (pg/mL) | 16.79 [9.371-27.33] | 22.59 ± 6.092 | 0 [0-0.3933] | 1.773 ± 1.606 | **0.002** | **0.0093** |
| VEGFA (pg/mL) | 156.3 [137.7-189.9] | 157.1 ± 12.82 | 108.2 [92.2-139.3] | 132.4 ± 24.88 | 0.1934 | 0.3385 |
| β-NGF (pg/mL) | 0 [0-0] | 0 ± 0 | 5.081 [4.325-15.36] | 10.14 ± 3.02 | **< 0.0001** | **0.0014** |
| **DED** | | | | | |  |
| OSDI | 32 [22-42.5] | 32.91 ± 2.276 | 11 [6-25] | 14.55 ± 1.882 | **<0.0001** | **0.0004** |
| TBUT (s) | 4 [3-6.5] | 5.515 ± 0.6814 | 7 [5-10] | 8.333 ± 0.7053 | **0.002** | **0.0056** |
| Corneal Staining (Oxford) | 0 [0-0.5] | 0.3333 ± 0.1205 | 0 [0-0.5] | 0.303 ± 0.1019 | 0.8244 | 0.9618 |
| Schirmer test (mm/5') | 9 [4-14] | 11.06 ± 1.496 | 10 [6.5-20.5] | 13.52 ± 1.555 | 0.0971 | 0.1510 |
| Tear osmolarity (mOsm/L) | 299 [291-311] | 301.4 ± 2.568 | 296 [288.5-304.5] | 297 ± 1.999 | **0.0333** | 0.0666 |
| conc. proteins (mg/dL) | 0.48 [0.37-0.62] | 0.55 ± 0.04 | 0.53 [0.44-0.72] | 0.61 ± 0.05 | 0.1752 | 0.2453 |
| GDF-15 (pg/mL) | 73.62 [45.14-110.5] | 77.6 ± 7.214 | 86.58 [68.19-115.3] | 95.46 ± 7.371 | **0.0112** | **0.0261** |
| OPN (pg/mL) | 7028 [0-24030] | 12723 ± 2518 | 11708 [1920-20199] | 15564 ± 3497 | 0.381 | 0.4849 |
| OPG (pg/mL) | 0 [0-0] | 4.033 ± 3.527 | 93.37 [82.25-116.4] | 101 ± 7.317 | **<0.0001** | **0.0004** |
| PDGF-AB (pg/mL) | 11.26 [0-56.58] | 28.52 ± 5.649 | 16.73 [0-48.26] | 30.65 ± 8.088 | 0.8693 | 0.9362 |
| PDGF-CC (pg/mL) | 639.7 [375.9-856.8] | 711.4 ± 76.61 | 737.9 [590.8-1165] | 1024 ± 130.8 | **0.0473** | 0.0828 |
| TNF-α (pg/mL) | 18.66 [8.85-27.31] | 19.26 ± 2.352 | 0 [0-0.2022] | 0.4737 ± 0.2681 | **<0.0001** | **0.0004** |
| VEGFA (pg/mL) | 143.5 [96.87-226.1] | 160.3 ± 13.91 | 151 [101.8-227.4] | 168.9 ± 15.33 | 0.943 | 0.9430 |
| β-NGF (pg/mL) | 0 [0-0] | 0 ± 0 | 5.363 [3.588-6.86] | 5.76 ± 0.55 | **<0.0001** | **0.0004** |
| **Sham** | | | | | |  |
| **Preclinical** | | | | | | |
| OSDI | 12 [11.25-12] | 11.2 ± 0.3449 | 16.5 [8-21.25] | 16.65 ± 2.617 | 0.059 | 0.1377 |
| TBUT (s) | 5.5 [4-9.75] | 6.5 ± 0.6469 | 6.5 [4-10] | 7.4 ± 0.8596 | 0.5852 | 0.7448 |
| Corneal Staining (Oxford) | 0 [0-0] | 0.25 ± 0.123 | 0 [0-0] | 0.275 ± 0.1332 | 0.8241 | 0.8241 |
| Schirmer test (mm/5') | 7 [3-13.25] | 8.35 ± 1.36 | 8 [5-15.75] | 10.3 ± 1.648 | 0.1523 | 0.2369 |
| Tear osmolarity (mOsm/L) | 296 [293.3-303.8] | 296.4 ± 2.411 | 306.5 [299.3-310] | 308 ± 4.079 | **0.0011** | **0.0039** |
| conc. proteins (mg/dL) | 0.43 [0.39-0.54] | 0.48 ± 0.04 | 0.5 [0.43-0.67] | 0.55 ± 0.04 | 0.1213 | 0.2123 |
| GDF-15 (pg/mL) | 79.81 [57.36-119.3] | 90.85 ± 10.6 | 88.13 [68.46-108.3] | 88.83 ± 7.427 | 0.6951 | 0.7486 |
| OPN (pg/mL) | 6513 [3167-19032] | 10889 ± 2337 | 13997 [6704-25953] | 23909 ± 7223 | 0.1044 | 0.2088 |
| OPG (pg/mL) | 0 [0-0] | 0.6495 ± 0.6495 | 90.96 [64.78-119.4] | 92.7 ± 7.912 | **<0.0001** | **0.0005** |
| PDGF-AB (pg/mL) | 16.8 [7.489-31.63] | 27.11 ± 7.609 | 20.52 [0-29.71] | 26.61 ± 8.151 | 0.6143 | 0.7167 |
| PDGF-CC (pg/mL) | 787 [597.5-1379] | 940.5 ± 107.2 | 594.5 [385.5-961] | 694.8 ± 93.38 | **0.0419** | **0.1173** |
| TNF-α (pg/mL) | 21.33 [11.12-25.17] | 22.04 ± 3.147 | 0 [0-0] | 0.87 ± 0.52 | **<0.0001** | **0.0005** |
| VEGFA (pg/mL) | 128.6 [79.08-190.5] | 145.6 ± 21.85 | 123.3 [80.19-153.1] | 126.8 ± 16.73 | 0.4222 | 0.5911 |
| β-NGF (pg/mL) | 0 [0-0] | 0 ± 0 | 5.16 [3.06-7.21] | 5.52 ± 0.71 | **<0.0001** | **0.0005** |
| **DED** | | | | | | |
| OSDI | 25 [19-35.5] | 28.24 ± 2.472 | 34 [23.5-40.5] | 32.28 ± 2.412 | 0.1002 | 0.2004 |
| TBUT (s) | 5 [4.5-11] | 7.4 ± 0.8165 | 5 [4-9] | 6.84 ± 0.8538 | 0.2354 | 0.4120 |
| Corneal Staining (Oxford) | 0 [0-1] | 0.34 ± 0.103 | 1 [0-1] | 0.68 ± 0.1381 | **0.021** | 0.0588 |
| Schirmer test (mm/5') | 7 [5-13.5] | 9.8 ± 1.718 | 7 [5-17] | 10.92 ± 1.881 | 0.531 | 0.6195 |
| Tear osmolarity (mOsm/L) | 295 [288-300] | 296.9 ± 3.723 | 302 [299-311] | 304.6 ± 2.134 | **0.0005** | **0.0018** |
| conc. proteins (mg/dL) | 0.49 [0.42-0.64] | 0.57 ± 0.05 | 0.5 [0.42-0.66] | 0.56 ± 0.04 | 0.8087 | 0.8087 |
| GDF-15 (pg/mL) | 69.75 [45.62-102] | 78.83 ± 9.331 | 78.47 [61.95-98.13] | 85.9 ± 8.107 | 0.4842 | 0.6163 |
| OPN (pg/mL) | 5351 [212.5-19608] | 9569 ± 2002 | 14114 [7731-22119] | 15750 ± 2216 | **0.0438** | 0.1022 |
| OPG (pg/mL) | 0 [0-0] | 4.587 ± 4.587 | 90.7 [77.64-127.1] | 98.95 ± 7.242 | **<0.0001** | **0.0005** |
| PDGF-AB (pg/mL) | 21.98 [5.931-64.65] | 41.57 ± 9.826 | 27.32 [4.177-44.85] | 30.74 ± 5.581 | 0.438 | 0.6132 |
| PDGF-CC (pg/mL) | 686.6 [464.9-1183] | 885.4 ± 113.4 | 662.1 [417.2-933.5] | 747.4 ± 81.75 | 0.3603 | 0.5605 |
| TNF-α (pg/mL) | 16.82 [8.33-29.83] | 19.91 ± 2.77 | 0 [0-0] | 0.24 ± 0.17 | **<0.0001** | **0.0005** |
| VEGFA (pg/mL) | 108.6 [70.85-159.1] | 120.4 ± 14.51 | 96.71 [60-170] | 129.5 ± 20.77 | 0.6668 | 0.7181 |
| β-NGF (pg/mL) | 0 [0-0] | 0 ± 0 | 4.75[4.19-7.29] | 5.43 ± 0.42 | **<0.0001** | **0.0005** |

**^‡^** Wilcoxon matched-pairs signed-rank test; OSDI, Ocular Surface Disease Index; TBUT, tear film break-up time; GDF-15, Growth differentiation factor 15; OPN, osteopontin; OPG, osteoprotegerin; PDGF, Platelet-Derived Growth Factor; TNF-α, Tumor Necrosis Factor alpha; VEGFA, Vascular Endothelial Growth Factor A; β-NGF, Nerve Growth Factor-beta; q-values represent p-values corrected for multiple comparisons using the Benjamini-Hochberg procedure.

**Supplementary Table 4. Dynamics of ocular parameters in patients undergoing cataract surgery who received low-level light therapy (LLLT), categorized based on final improvement outcome.**

| **LLLT** | **T0** | | **T1** | | **p-value^‡^** | **q-value** |
| --- | --- | --- | --- | --- | --- | --- |
|  | **Median [CI]** | **mean ± s.e.m.** | **Median [CI]** | **mean ± s.e.m.** |  |  |
| ***No clinical improvement*** | | | | | | |
| OSDI | 22.5 [10.25-42.75] | 26.25 ± 3.675 | 17.5 [6.5-28] | 17.63 ± 2.484 | **0.0101** | **0.0283** |
| TBUT (s) | 4 [3-5.75] | 4.917 ± 0.5833 | 7 [5-9.75] | 8.292 ± 0.9771 | **0.0001** | **0.0004** |
| Corneal Staining (Oxford) | 0 [0-0] | 0.125 ± 0.06896 | 0 [0-0] | 0.2083 ± 0.08468 | 0.484 | 0.5647 |
| Schirmer test (mm/5') | 7 [4-12.75] | 10.5 ± 1.835 | 7.5 [5-14] | 11.29 ± 1.769 | 0.7274 | 0.7834 |
| Tear osmolarity (mOsm/L) | 296.5 [288.5-304.3] | 296.1 ± 2.116 | 295 [287.3-301.5] | 293.9 ± 2.37 | 0.212 | 0.3298 |
| GDF-15 (pg/mL) | 68.54 [51.29-109.4] | 77.12 ± 7.998 | 87.1 [63.38-112.7] | 92.16 ± 8.354 | 0.0946 | 0.2207 |
| OPN (pg/mL) | 7304 [34.5-14890] | 12007 ± 3596 | 11198 [2336-20953] | 28024 ± 13127 | 0.2803 | 0.3924 |
| OPG (pg/mL) | 0 [0-0] | 6.112 ± 4.941 | 92.98 [82.3-134] | 115.3 ± 14.8 | **<0.0001** | **0.0005** |
| PDGF-AB (pg/mL) | 13.82 [0-48.02] | 32.13 ± 9.559 | 16.7 [0-40.11] | 34.36 ± 12.64 | 0.3849 | 0.4899 |
| PDGF-CC (pg/mL) | 715.7 [498.3-1179] | 882.6 ± 110.6 | 1004 [664.6-1247] | 1114 ± 167.2 | 0.0946 | 0.1892 |
| TNF-α (pg/mL) | 17.28 [11.13-25.01] | 21 ± 3.337 | 0 [0-0] | 0.8067 ± 0.6711 | **<0.0001** | **0.0005** |
| VEGFA (pg/mL) | 156.3 [126.2-216.5] | 172.1 ± 12.56 | 126.1 [93.78-168.7] | 146.1 ± 15.82 | 0.1128 | 0.1974 |
| β-NGF (pg/mL) | 0 [0-0] | 0 ± 0 | 5.43 [4.43-7.1] | 7.486 ± 1.346 | **<0.0001** | **0.0005** |
| ***Clinical improvement*** | | | | | | |
| OSDI | 31 [19-38] | 28.53 ± 2.74 | 6 [3-11] | 6.158 ± 0.9217 | **0.0001** | **0.0004** |
| TBUT (s) | 4 [3-9] | 6.421 ± 1.08 | 10 [6-12] | 8.842 ± 0.9801 | **0.0228** | 0.0532 |
| Corneal Staining (Oxford) | 0 [0-1] | 0.4211 ± 0.1922 | 0 [0-0] | 0.3158 ± 0.1539 | 0.5716 | 0.6156 |
| Schirmer test (mm/5') | 8 [4-13] | 9.368 ± 1.475 | 10 [7-23] | 13.58 ± 2.098 | **0.0345** | 0.0604 |
| Tear osmolarity (mOsm/L) | 309 [293-318] | 306.8 ± 3.88 | 299 [286-309] | 297.3 ± 2.842 | **0.0055** | **0.0154** |
| GDF-15 (pg/mL) | 90.45 [44.57-112] | 83.44 ± 10.26 | 100.6 [74.83-125.7] | 107.7 ± 10.87 | **0.0255** | 0.0510 |
| OPN (pg/mL) | 9140 [0-28049] | 14568 ± 3658 | 13806 [1848-31341] | 21504 ± 6056 | 0.3388 | 0.4312 |
| OPG (pg/mL) | 0 [0-0] | 0.8545 ± 0.7255 | 109.8 [82.24-131.7] | 110.1 ± 11.25 | **0.0001** | **0.0004** |
| PDGF-AB (pg/mL) | 21.74 [1.344-64.2] | 32.58 ± 7.408 | 20.38 [5.016-55.82] | 41.57 ± 12.83 | 0.5713 | 0.6665 |
| PDGF-CC (pg/mL) | 630 [324.4-851.7] | 729.1 ± 134.5 | 737.9 [516.3-1337] | 933.4 ± 115.8 | 0.2352 | 0.3659 |
| TNF-α (pg/mL) | 18.76 [5.511-31.22] | 18.82 ± 2.997 | 0 [0-0.8877] | 0.7367 ± 0.4595 | **0.0001** | **0.0004** |
| VEGFA (pg/mL) | 123.2 [82.66-171.4] | 143.7 ± 19 | 142 [109.2-256.6] | 178.5 ± 21.96 | 0.3242 | 0.4539 |
| β-NGF (pg/mL) | 0 [0-0] | 0 ± 0 | 4.73 [3.26-7.18] | 5.88 ± 0.87 | **<0.0001** | **0.0004** |

**^‡^** Wilcoxon matched-pairs signed-rank test; OSDI, Ocular Surface Disease Index; BUT, tear film break-up time; GDF-15, Growth differentiation factor 15; OPN, osteopontin; OPG, osteoprotegerin; PDGF, Platelet-Derived Growth Factor; TNF-α, Tumor Necrosis Factor alpha; VEGFA, Vascular Endothelial Growth Factor A; β-NGF, Nerve Growth Factor-beta; q-values represent p-values corrected for multiple comparisons using the Benjamini-Hochberg procedure.

**Supplementary Table 5. ROC analysis of predictive mathematical models for clinical improvement following received low-level light therapy (LLLT).**

| **Mathematical model** | **AUC** | **S.E.** | ***p-*value** | **95% Confidence Interval** |
| --- | --- | --- | --- | --- |
| **Model_1** | 0.840 | 0.059 | **<0.001** | 0.724-0.956 |
| **Model_1_covariates** | 0.922 | 0.042 | **<0.001** | 0.840-1.000 |
| **Model_2** | 0.771 | 0.072 | **0.003** | 0.630-0.913 |
| **Model_2_covariates** | 0.977 | 0.019 | **<0.001** | 0.940-1.000 |

Model_1: basal osmolarity, GDF-15, OPN, TNFα, PDGF-CC; covariates: age, gender, basal clinical status

Model_2 (at T1): osmolarity, GDF-15, β-NGF, TNFα, VEGFA; covariates: age, gender, clinical status

**Supplementary Table 6.** **Multivariate regression analysis evaluating baseline (T0) tear biomarkers as predictors of clinical improvement following LLLT.**

| **Parameter** |  |  | **Multivariate analysis** | | |
| --- | --- | --- | --- | --- | --- |
|  | **B** | **S.E.** | **HR** | **95% CI** | ***p*-value** |
| **Osmolarity** | 0.085 | 0.036 | 1.089 | 1.014-1.170 | **0.019** |
| GDF-15 | 0.044 | 0.023 | 1.045 | 0.999-1.092 | 0.054 |
| OPN | 0.0001 | 0.0001 | 1.000 | 1.000-1.000 | 0.040 |
| **TNF-α** | -0.234 | 0.098 | 0.791 | 0.653-0.959 | **0.017** |
| PDGF-CC | -0.002 | 0.001 | 0.998 | 0.996-1.000 | 0.103 |

**Supplementary Table 7.** **Multivariate regression analysis evaluating tear biomarkers at T1 (one month after cataract surgery) in relation to LLLT-associated clinical improvement.**

| **Parameter** |  |  | **Multivariate analysis** | | |
| --- | --- | --- | --- | --- | --- |
|  | **B** | **S.E.** | **HR** | **95% CI** | ***p*-value** |
| Osmolarity | 0.037 | 0.031 | 1.038 | 0.977-1.102 | 0.229 |
| GDF-15 | -0.224 | 0.122 | 0.799 | 0.630-1.014 | 0.066 |
| **β-NGF** | 0.032 | 0.016 | 1.032 | 1.001-1.065 | **0.046** |
| TNF-α | 0.050 | 0.188 | 1.051 | 0.727-1.518 | 0.792 |
| VEGFA | 0.004 | 0.004 | 1.004 | 0.996-1.013 | 0.320 |
