# Supplementary material for: Tear biomarker changes and ocular surface recovery with low-level light therapy after cataract surgery: a double-masked randomized controlled clinical trial
Source: Sci Rep. 2026 May 20;16:22977. doi: 10.1038/s41598-026-53521-4 (PMC13391487; doi:10.1038/s41598-026-53521-4)
Supplement: Supplementary file 2 — Supplementary Material 2 [file 41598_2026_53521_MOESM2_ESM.pdf]

Supplementary Figure 1

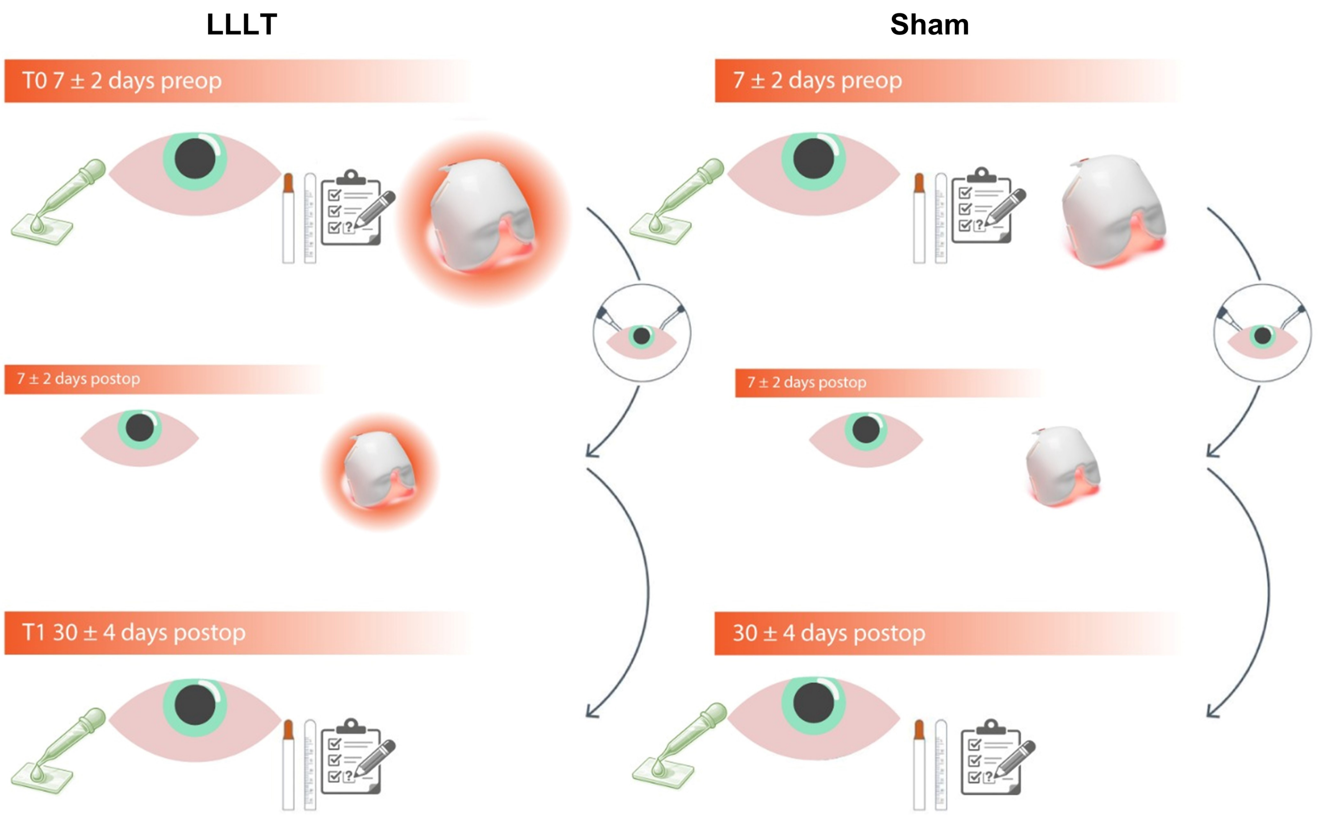

Supplementary Figure 1. Schematic overview of the study design. Patients were randomly assigned to receive two sessions of LLLT or sham treatment (one week before and one week after cataract surgery). At baseline, prior to any treatment, patients underwent ocular surface assessment and tear film collection for biomarker analysis. Follow-up evaluation, including ocular surface examination and tear biomarker measurement, was performed 30 days after cataract surgery.
