# Supplementary material for: Tear biomarker changes and ocular surface recovery with low-level light therapy after cataract surgery: a double-masked randomized controlled clinical trial
Source: Sci Rep. 2026 May 20;16:22977. doi: 10.1038/s41598-026-53521-4 (PMC13391487; doi:10.1038/s41598-026-53521-4)
Supplement: Supplementary file 3 — Supplementary Material 3 [file 41598_2026_53521_MOESM3_ESM.pdf]

Supplementary Figure 2: CONSORT 2025 Flow Diagram

Flow diagram of the progress through the phases of a randomised trial of two groups: enrolment, intervention allocation, follow-up, and data analysis.

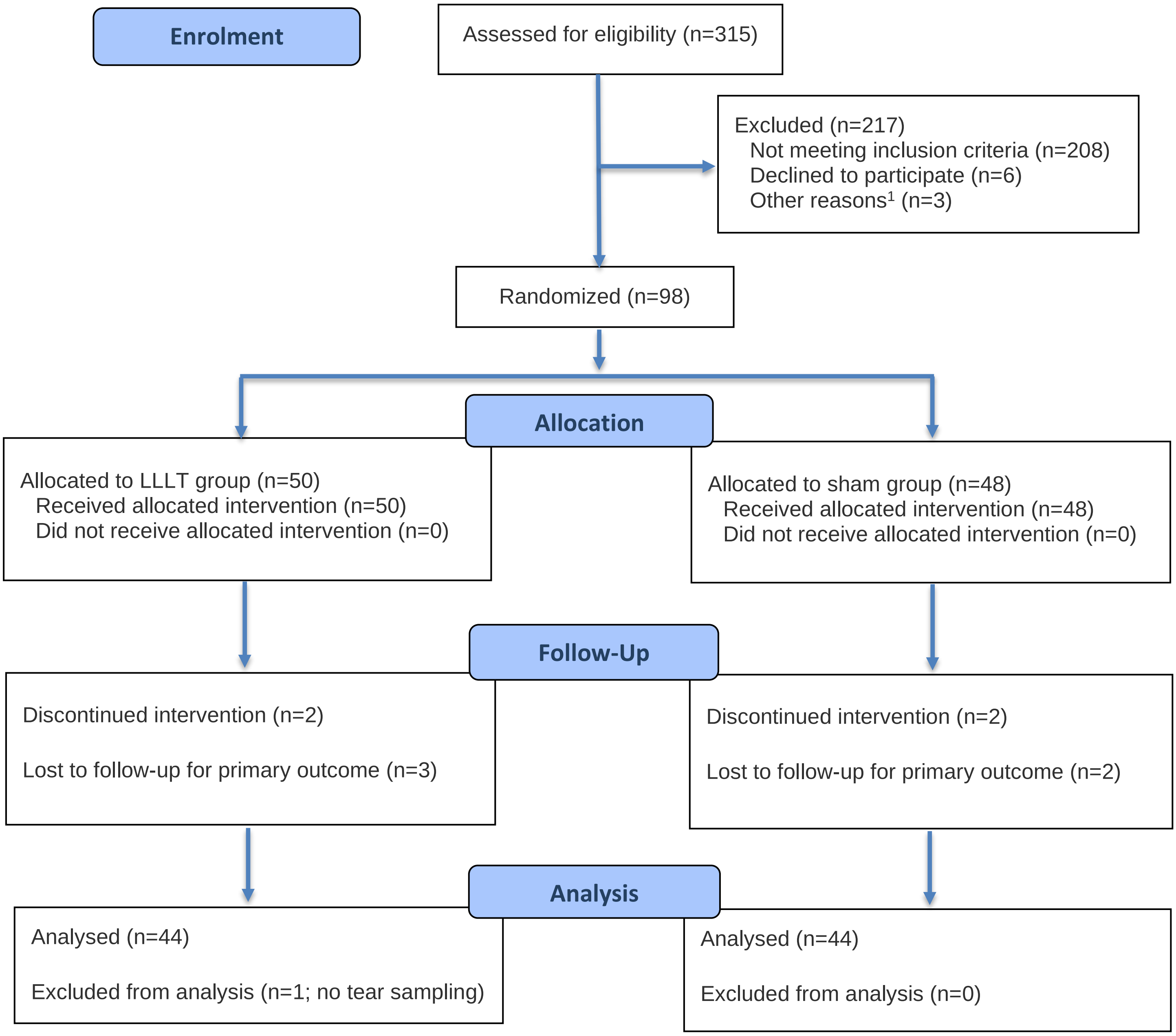

Note: [1] Other reasons - included patients deemed temporarily unfit for surgery due to lack of cardiology clearance or laboratory findings suggestive of an acute systemic condition, requiring postponement and further medical evaluation.

Citation: Hopewell S, Chan AW, Collins GS, Hróbjartsson A, Moher D, Schulz KF, et al. CONSORT 2025 Statement: updated guideline for reporting randomised trials. BMJ. 2025; 388:e081123. https://dx.doi.org/10.1136/bmj-2024-081123
© 2025 Hopewell et al. This is an Open Access article distributed under the terms of the Creative Commons Attribution License (https://creativecommons.org/licenses/by/4.0/), which permits unrestricted use, distribution, and reproduction in any medium, provided the original work is properly cited.
