# Supplementary material for: Tear biomarker changes and ocular surface recovery with low-level light therapy after cataract surgery: a double-masked randomized controlled clinical trial
Source: Sci Rep. 2026 May 20;16:22977. doi: 10.1038/s41598-026-53521-4 (PMC13391487; doi:10.1038/s41598-026-53521-4)
Supplement: Supplementary file 4 — Supplementary Material 4 [file 41598_2026_53521_MOESM4_ESM.docx]

**Research protocol**

1. **Project summary**

Dry eye disease (DED) is a common complication following cataract surgery, potentially impairing visual recovery. Low-level light therapy (LLLT) has emerged as a noninvasive approach to improve ocular surface status. This study evaluated the perioperative effects of LLLT on ocular surface modulation by correlating clinical outcomes with tearbiomarker dynamics and developing predictive models to identify patients most likely to benefit from LLLT. Ocular surface evaluations were performed at various scheduled time points: baseline, 1 week prior to surgery before the first treatment/sham session (T0), and 1 month following cataract surgery (here defined as T1). As for our methods, eighty-eight patients undergoing cataract surgery and randomised to receive LLLT (n=44) or sham treatment (n=44) were included in the study analysis.  Clinical evaluation, including Ocular Surface Disease Index questionnaire, tear breakup time, Schirmer test, and tear osmolarity, was performed preoperatively and one month postoperatively to classify patients in subclinical or clinical DED. Tear levels of biomarkers involved in tissue repair and neurotrophic-signalling (GDF-15, β-NGF, VEGFA, PDGF-AB, PDGF-CC) and inflammation (OPN, OPG, TNF-α) were quantified using Luminex FlexMap3D technology. The results showed that LLLT-treated patients showed significantly greater clinical improvement compared to sham controls (44.2% vs. 4.4%; p<0.0001). LLLT significantly increased GDF-15 and PDGF-CC, particularly in patients with baseline DED, while β-NGF increased mainly in preclinical patients. Inflammatory biomarker profiles indicated resolution in the LLLT group, whereas sham-treated patients showed persistent inflammatory patterns.

1. **General information**

**Effect of Low-Level Light Therapy on Ocular Surface Parameters in Patients Undergoing Cataract Surgery: A Randomized Controlled Clinical Trial (Organization's Unique Protocol ID Grigore T. Popa n.54/290724)**

The study received funding from a project under The Health Program (PS) 2021–2027, Policy Objective 1, Priority 5, project title “Development of translational research for vaccines, serums and other biological drugs—Acronym CANTAVAC 2.0”, SMIS code 326920.

1. **Investigators**

Mihaela-Madalina Timofte-Zorila ^1,2^, Mariana Pavel-Tanasa ^1,3,^*, Giuseppe Giannaccare ^4^, Nicoleta Vlas ^1,2^, Filippo Lixi ^4^, Mario Troisi ^5, 6^, Daniela Constantinescu ^1,3^, Radu Tanasa ^7^, Diana Alecu ^1,2^, Sabina Turcas ^1^, Sinziana Istrate ^8,9^, Daciana Elena Branisteanu ^1,10^, Cristina Preda ^1^, and Daniel Constantin Branisteanu ^1,2^

^1^ Grigore T. Popa University of Medicine and Pharmacy Iasi, 700115 Iasi, Romania

^2^ Ophthalmology Clinic, Cai Ferate Clinical Hospital, 700506 Iasi, Romania

^3^ Laboratory of Immunology, St. Spiridon County Clinical Hospital, 700111 Iasi, Romania

^4^ Eye Clinic, Department of Surgical Sciences, University of Cagliari, 09124 Cagliari, Italy

^5^ Eye Clinic, Department of Neurosciences, Reproductive and Odontostomatological Sciences, Federico II University, 80131 Naples, Italy

^6^  Ophthalmologic Unit, Salerno University Hospital, 84131 Salerno, Italy

^7^ Faculty of Physics, Alexandru Ioan Cuza University, 700506 Iasi, Romania

^8^  Carol Davila University of Medicine and Pharmacy, 050474 Bucharest, Romania

^9^ Optissima, Barbu Văcărescu 102, 020283, Bucharest, Romania

^10^ Dermatology Clinic, Cai Ferate Clinical Hospital, 700506 Iasi, Romania

***Correspondence to:**

**Mariana Pavel-Tanasa**, [mariana.pavel-tanasa@umfiasi.ro](mailto:mariana.pavel-tanasa@umfiasi.ro)

***Mariana Pavel-Tanasa, MD, PhD***

Associate Professor of Immunology

Faculty of Medicine, *Grigore T Popa* University of Medicine and Pharmacy Iasi, Romania

Head of Immunology Laboratory

Laboratory of Immunology, *Sf. Spiridon* County Clinical Emergency Hospital, Iasi, Romania

[mariana.pavel-tanasa@umfiasi.ro](mailto:mariana.pavel-tanasa@umfiasi.ro); <https://orcid.org/0000-0002-2802-5993>

1. **Rationale & background information**

Nowadays, cataract surgery is one of the most frequently performed surgical procedures worldwide, with excellent visual outcomes in the vast majority of cases. Despite its favourable prognosis in terms of clinical results and postoperative recovery, cataract surgery may be associated with several complications, among which postoperative dry eye disease (DED) is one of the most common. (Day et al., 2015).

In-office instrumental therapies represent a new option in the armamentarium of DED treatment. Among these, low-level light therapy (LLLT), also termed photobiomodulation or phototherapy, has recently gained increased attention for both managing and preventing DED in the setting of ocular surgery (Hamblin, 2016a). This therapy is based on the use of various nonionizing forms of light in the visible and near-infrared (NIR) spectrum from sources like lasers, light-emitting diodes (LED), and filtered lamps able to activate a cascade of complex photophysical and photochemical events at various biological scales (Anders et al., 2019; Hamblin, 2016b).

These molecular events result in cellular responses that promote migration, proliferation, anti-inflammatory activity, and tissue repair via transcription factor activation and regulation of cytokines and growth factors (Cannas et al., 2024; De Freitas and Hamblin, 2016; Viggiano et al., 2025; Yadav and Gupta, 2017).

1. **Study goals and objectives**

The aim of the present study was to evaluate the effect of perioperatively administered LLLT on ocular surface status by correlating clinical parameters with biological changes measured through tear film biomarkers. Evaluating these parameters before and after cataract surgery at various time points might allow the identification of prognostic factors able to predict which patients are most likely to benefit from LLLT. Such an integrated clinical–molecular approach would be crucial for determining whether LLLT provides a better modulation of ocular surface status compared to standard postoperative care alone in terms of inflammatory regulation, neurotrophic support, and tissue repair.

1. **Study design**

A prospective, double-masked randomized clinical, sham-controlled study was carried out at the Cai Ferate Clinical Hospital in Iasi, Romania. The study protocol complied in accordance with the 1964 Helsinki Declaration and its later amendments, and was approved by the local Ethics Committee of the Cai Ferate Clinical Hospital of Iasi, Romania (approval number: 54/29.07.2024) and retrospectively registered at ClinicalTrials.gov (NCT07067294) on 05.07.2025. No patients were involved in setting the research questions or the outcome measures, nor were they involved in developing plans for recruitment, design, or implementation of the study. No patients were asked to advise on interpretation or writing up of results. Prior to participation, all patients provided written informed consent.

Patients aged 60 years or older scheduled to undergo routine cataract surgery by phacoemulsification were screened for eligibility criteria and recruited in the study between January and March 2025.

Exclusion criteria were: i) previous ocular surgery in either eye, or any concomitant ocular pathology; ii) use of topical ocular medications (such as anti-inflammatory agents, artificial tears or immunomodulatory therapy) or device-based treatments; iii) use of systemic medications associated with tear film alterations (including antihistamines, diuretics, antidepressants or hormone replacement therapy); iv) use of contact lens in the last month; v) autoimmune diseases (e.g., Sjögren’s syndrome); vi) intraoperative or postoperative complications potentially affecting ocular surface integrity.

The sample size was determined a priori based on the primary outcome, defined as the change in Ocular Surface Disease Index (OSDI) score between the treatment and sham groups. The calculation used a two-sided test for comparing mean changes between groups, with a significance level (α) of 0.05 and a statistical power of 80%. A clinically meaningful difference of 8 points in OSDI score was assumed, consistent with previously reported minimal clinically important differences [12–14]. Assuming a standard deviation of 12 points and a pre–post correlation of 0.5, the required sample size was estimated at 36 patients per group. To account for potential dropouts and preserve statistical power, 98 participants were initially enrolled. After attrition due to loss to follow-up or discontinuation of the intervention, 45 patients remained in the LLLT group and 44 in the sham group. One participant in the LLLT group declined tear film sample collection for inflammatory biomarker analysis. Consequently, 88 participants were included in the final analysis.

The other study outcomes were structured into distinct categories to ensure methodological clarity. Secondary clinical endpoints comprised tear film breakup time (TBUT), corneal fluorescein staining, the Schirmer I test, and tear osmolarity. The evaluation of inflammatory and reparative biomarkers was designated as exploratory biomarker endpoints. To further investigate these relationships, post hoc predictive analyses were performed to identify associations between baseline profiles and clinical responses.

No significant changes were made to the study protocol after the study has started. Eligible participants were randomly assigned to each the active low-level light treatment group or the sham-control group. Randomization was performed using a computerized allocation sequence (http://www.sealedenvelope.com). Allocation concealment was ensured through sequentially numbered, opaque, sealed envelopes. Both patients and investigators responsible for clinical evaluations remained unaware of group assignment throughout the study period

### **Methodology**

Before surgery, the ocular surface status of each participant was characterized using a combined assessment of patient-reported symptoms and objective tear film tests. Clinical symptom severity was quantified using the Ocular Surface Disease Index questionnaire (OSDI). Tear film stability was evaluated by measuring tear break-up time (TBUT), while basal and reflex tear secretion were assessed using the Schirmer I test. Based on these parameters, patients were categorized into 3 groups: i) normal (OSDI score <13, TBUT >10 seconds, and Schirmer I ≥10 mm); ii) preclinical (OSDI score <13 plus at least one between TBUT <10 seconds or Schirmer I <10 mm), and iii) DED (OSDI score ≥13plus at least one between TBUT <10 seconds or Schirmer I <10 mm).

Participants allocated to the treated group underwent periocular LLLT using the Eye-light^®^ device (EspansioneMarketing S.p.A., Bologna, Italy), which emits red light of 633 ± 10 nm wavelength. Two therapy sessions were administered for 15 minutes each, 7 days prior to cataract surgery and 7 days after surgery.

The control group received a sham procedure using the same device set to demo mode, delivering less than 30% of the therapeutic light intensity. This approach ensured patient masking while avoiding biologically meaningful light exposure.

All cataract procedures were performed by a single experienced surgeon employing a standardized micro-incisional phacoemulsification technique (DCB). Eyes with any intraoperative or postoperative aggravations were excluded from further analysis. All patients followed an equivalent postoperative treatment protocol. This included a fixed combination of topical dexamethasone and netilmicin administered for 1 week, followed by a gradual taper of dexamethasone monotherapy over the subsequent 2 weeks. In addition, topical ketorolac was prescribed 3 times daily for 1 month. Preservative-free artificial tears containing cross-linked hyaluronic acid, trehalose, and sterilamine encapsulated in liposomes were administered 3 times daily for 3 months after the surgery.

Tear samples were collected using Schirmer type I strips (Haag-Streit, UK) without topical anesthesia, following our previously reported protocol (M. M. Timofte-Zorila et al., 2025). The strips were placed in the lower conjunctival fornix for up to 5 minutes, then individually stored in sterile microtubes at −80 °C. For protein extraction, the strips were thawed and incubated in 400 μL of 1.5 M Tris–HCl buffer (pH 8.8) containing protease inhibitors for 3 hours, followed by centrifugation at 16,000× g for 15 minutes (4 °C). Total protein content was determined by Nanodrop (ThermoFischerScientific), and tear cytokines and chemokines were quantified using a multiplex bead-based immunoassay (R&D Systems) on a Luminex FlexMap3D platform.

Tear biomarkers were quantified using a customised Luminex multiplex assay enabling the simultaneous measurement of growth differentiation factor 15 (GDF-15), β-nerve growth factor (β-NGF), vascular endothelial growth factor A (VEGF-A), platelet-derived growth factors (PDGF-AB and PDGF-CC), osteopontin (OPN), osteoprotegerin (OPG), and tumour necrosis factor alpha (TNF-α) in accordance with the manufacturer’s recommendations. Briefly, thawed tear samples were diluted 1:2 using the supplied sample diluent. Lyophilized analyte standards were reconstituted with calibrator diluent and combined to generate the highest concentration standard, followed by a three-fold serial dilution to obtain 6 standard points. The upper standard concentrations were 4,060 pg/mL for GDF-15, 940 pg/mL for β-NGF, 2,120 pg/mL for VEGF-A, 6,140 pg/mL for PDGF-AB, 16,990 pg/mL for PDGF-CC, 258,220 pg/mL for OPN, 15,500 pg/mL for OPG, and 1,680 pg/mL for TNF-α. Assay procedures were otherwise performed as previously described (M. M. Timofte-Zorila et al., 2025), with data acquisition carried out on a Luminex FlexMap3D system. Final biomarker concentrations were normalised to total tear protein content as determined by NanoDrop analysis.

1. **Follow-up**

Ocular surface evaluations were performed at various scheduled time points: baseline, 1 week prior to surgery before the first treatment/ sham session (T0), and 1 month following cataract surgery (T1). At each visit, a standardized, non-invasive ocular surface examination was conducted. Outcomes included the OSDI score, TBUT, tear osmolarity (TearLab™ system), Schirmer I test, and ocular surface staining (Oxford scale). At both T0 and T1, tear samples were also collected for biomarker analysis. All assessments were carried out under the same environmental conditions by the same masked examiner to ensure methodological consistency and minimize bias. The study timeline is summarized in Supplementary Figure 1. At T1, clinical improvement was defined as a transition from DED to borderline conditions or normal status, or from borderline condition to normal status.

1. **Data Management and Statistical Analysis**

Statistical analyses were conducted using GraphPad Prism version 5 (GraphPad Software, San Diego, CA, USA) and SPSS version 27 (IBM SPSS Statistics, Chicago, IL, USA). Data are generally presented as box-and-whisker plots illustrating minimum and maximum values. Tear biomarker distributions were initially assessed for normality and homogeneity of variance using the Shapiro–Wilk test. One patient who withdrew consent for tear sampling was excluded from the biomarker analyses, as tear collection could not be performed. Variables that met the assumptions of normality were further analyzed using two-way repeated-measures ANOVA, followed by Bonferroni post hoc correction for multiple comparisons. As most variables did not satisfy normality criteria, non-parametric statistical methods were applied: group comparisons were performed using the Kruskal–Wallis test with Dunn’s multiple-comparison post hoc analysis, while paired non-parametric data were evaluated using the Wilcoxon matched-pairs signed-rank test. Comparisons between two independent patient subgroups were conducted using the Mann–Whitney U test. Spearman’s rank correlation coefficients were calculated for non-parametric correlation analyses. Linear regression plots display the best-fit regression line together with the 95% confidence interval. Model fit was assessed using the coefficient of determination (R²), and statistical significance was determined by the F-test. For the analysis of tear film biomarkers, adjustments for multiple comparisons were performed to control the False Discovery Rate (FDR). Specifically, the Benjamini–Hochberg procedure was applied to account for the multiplicity of tests and reduce the likelihood of Type I errors; corresponding q-values were calculated in Python (version 3.12) for each biomarker across the study groups. Heat map visualizations were generated using OriginPro2024 software and were intended solely to provide a graphical overview of potential associations among tear biomarkers. Multivariate linear regression models incorporating clinical and paraclinical variables were developed to construct predictive mathematical models, whose ability to predict clinical improvement was evaluated using receiver operating characteristic (ROC) curve analysis in SPSS. To refine the predictive models and mitigate the risk of overfitting, we employed a LASSO (Least Absolute Shrinkage and Selection Operator) logistic regression analysis, which was implemented in Python (version 3.12) using the scikit-learn library. To ensure all biomarkers contributed equally to the regularization process regardless of their original units, all features were standardized using a StandardScaler prior to model fitting. The optimal regularization strength was determined through a stratified k-fold cross-validation procedure, using the SAGA solver and optimizing for the Area Under the Receiver Operating Characteristic curve (AUC). To maintain the reproducibility of our findings, a fixed random seed (random_state=10) was applied. Statistical significance was defined as a p-value < 0.05.

1. **1Quality assurance**

The study was conducted in compliance with ICH-GCP, the Declaration of Helsinki, and applicable national regulations. A risk-based quality management approach was implemented, including investigator training, standardized procedures, and ongoing clinical monitoring. Monitoring focused on critical processes such as informed consent, eligibility, protocol compliance, safety reporting, and data accuracy. An independent Data and Safety Monitoring Board (DSMB) reviewed accumulating safety data throughout the study. Data management was performed using validated electronic systems with restricted access, audit trails, and predefined data validation checks. All data were anonymized and managed in accordance with GDPR (EU 679/2016).

1. **Expected outcomes of the study**

The expected outcomes of the study were the changes in Ocular Surface Disease Index (OSDI) score, tear film stability (TBUT), Schirmer I test, corneal staining, tear osmolarity, and tear inflammatory and reparative biomarkers. The study also evaluated the safety and tolerability of the LLLT intervention.This is the first prospective study assessing the role of perioperative LLLT in mitigating post-cataract ocular surface disease in consecutive patients. The findings may support the integration of LLLT in perioperative ocular surface management protocols.

1. **1Dissemination of results and publication policy**

The preliminary results (at 1 month) were published at doi: 10.1007/s40123-025-01228-6. Epub 2025 Aug 24. PMID: 40849606; PMCID: PMC12413350.

**13. Problems anticipated**

**1.Patient-related errors**

- refusal to sign the informed consent form; errors due to loss to follow-up;
- errors related to the reporting of certain symptoms by patients, which cannot be quantifiedobjectively.
- losing follow-up

These types of difficulties can be overcome through effective communication with the patients included in the study, by explaining the potential benefits and possible risks in a way that each patient can understand, as well as the details regarding the confidentiality of data use.

**2. Errors related to research team members/equipment**

Errors that may occur during diagnosis are:

- errors in data recording or lack of data recording;
- errors due to manipulation through: contamination, mixing of samples and coding errors
- reading device errors;
- errors in interpreting analyses;

These types of difficulties can be overcome by careful recording and handling of samples.

Other types of errors that may occur in the LUMINEX tear film test are:

- low working volume

These types of errors can be prevented by choosing kits with adequate sensitivity, taking moistened strips up to 15 cm long, and noting the time required to moisten the Schirmer strip.

1. **Duration of the project**

The project has started on 8th January 20205 and was finished on the 11th September 2025. During that time, the data collection was made from January to March, the data analysis and the results at 1 month were done from July to August. Tha final probe analysis was done in December 2025

1. **1Safety considerations**

This protocol is unique compared to standard post-refractive surgery care because it targets cellular mitochondrial activity to enhance tissue repair and anti-inflammatory effects. It is non-pharmacological, non-contact, and well-tolerated, making it suitable for perioperative application. The light is delivered through closed eyelids, ensuring safety and comfort, and the device used is CE-marked for ophthalmic applications.

1. **Ethics**

The study protocol complied in accordance with the Declaration of Helsinki and was approved by the local Ethics Committee of “Cai Ferate” Clinical Hospital in Iasi, Romania (approval number: 54/29.07.2024), and all patients provided written informed consent.

1. **Project management**

Mihaela-Madalina Timofte-Zorila: Conceptualization, Methodology, Validation, Formal analysis, Investigation, Writing – Original Draft;

Mariana Pavel-Tanasa: Conceptualization, Software, Validation, Formal analysis, Data curation, Visualisation, Writing – Original Draft, Funding acquisition;

Giuseppe Giannaccare: Conceptualization, Methodology, Writing – Review & Editing;

Nicoleta Vlas: Formal analysis, Investigation; Writing – Review & Editing;

Filippo Lixi: Methodology, Writing – Review & Editing;

Mario Troisi: Methodology, Writing – Review & Editing;

Daniela Constantinescu: Formal analysis, Investigation, Resources;

Radu Tanasa: Software, Validation, Formal analysis, Data curation, Visualisation;

Diana Alecu: Formal analysis; Investigation, Writing – Review & Editing;

Sabina Turcas: Formal analysis; Investigation, Writing – Review & Editing;

Sinziana Istrate: Formal analysis; Investigation;

Daciana Elena Branisteanu: Data curation, Supervision;

Cristina Preda: Data curation, Supervision;

Daniel Constantin Branisteanu: Methodology, Writing – Review & Editing, Supervision, Funding acquisition.

1. **Informed consent forms**

Please read this form carefully and ask for further explanations if necessary, in order to give your consent to participate in the research entitled:

“**Effect of Low-Level Light Therapy on Ocular Surface Parameters in Patients Undergoing Cataract Surgery**”.

Your final written consent is required to participate in the aforementioned study.

1.  PURPOSE OF THE STUDY:

The study you are invited to participate in aims to identify compounds in the tear film involved in the onset and progression of ocular surface changes. These can cause discomfort manifested by stinging, excessive tearing, foreign body sensation in the eye, and photophobia (light sensitivity). Treatment following these investigations will be more effective and personalized, and you may benefit from low-level laser therapy (LLLT) to reduce eye inflammation.

2. PROCEDURES

We will collect data from your medical file regarding the diseases you suffer from, endocrinological evaluation if you are known to have these problems, as well as data on demographic characteristics (gender, age at diagnosis, number of years since diagnosis). An ophthalmological evaluation will be performed (vision assessment, non-contact intraocular pressure measurement, endothelial cell count, tear film break-up time, tear film osmolarity, Schirmer 1 test, tear film meniscus height, exophthalmometry, color perception, optical coherence tomography investigations). We invite you to respond anonymously to a questionnaire on the symptoms associated with dry eye syndrome (Ocular Surface Disease Index- OSDI). We will take strips of sterile paper with tears from the lower eyelid of both eyes to analyze their composition. The ophthalmological consultation will include the evaluation of the amount of tear secretions with these strips, so no additional sampling will be necessary for the present study. The study will be conducted over a period of 3 years at the Ophthalmology Clinic of the Clinical Hospital Cai Ferate, Iasi and involves multiple assessments necessary for the control of ophthalmological pathology, which will be recorded as disease progression within the study. We will monitor the health of the ocular surface in accordance with the recommended treatment, using all the methods described above.

In this study, participants will receive either active low-level light therapy (LLLT) for 15 minutes with their eyes closed. The treatment may be at 100% intensity, or it may be a sham treatment at low intensity (30%—no direct therapeutic effect) in order to compare the results. Assignment to the treatment group or the sham group will be random (by computerized lottery). Neither you nor your doctor will know in advance which treatment you are receiving.

3.    POSSIBLE BENEFITS

Identifying the composition of the tear film and the inflammatory factors involved in the onset and progression of ocular surface disease will allow for the optimization of topical (local) ophthalmic treatment with a therapeutic response superior to current therapeutic options and an improvement in quality of life.

If, following the investigations, a change in treatment is necessary, the costs will be covered by the Ministry of Health in accordance with the legislation in force, to the extent that you are eligible.

You will be able to benefit from photobiomodulation therapy through the application of a red light eye mask that has proven results in reducing the symptoms of induced dry eye. This treatment is applied according to the protocol recommended by the ophthalmologist and is free of charge.

There is no direct benefit for you in participating in this study, but you have the opportunity to benefit from free treatments and ophthalmological and immunological re-evaluation, with the optimization of a personalized treatment plan.

Indirect benefits for the scientific community: The study you are participating in will help improve knowledge about these changes in the tear film in patients with dry eye syndrome and those with endocrine disorders. These findings will open up new therapeutic options through the possible recommendation of biomarker testing in current clinical practice and treatment optimization.

The study you are participating in will help improve knowledge about changes in the tear film in patients with dry eye syndrome and will open up new therapeutic options through the possible recommendation of LLLT in current practice.

4.    POSSIBLE RISKS

The research involves minimal risk in terms of ophthalmological evaluation because all evaluations are non-contact (no touching of the eye surface), except when collecting tear film. Placing the strip on the lower eyelid to measure and collect tears may cause local discomfort in the eyes, a foreign body sensation, hyperlacrimation, and slight temporary local redness of the ocular surface.

To reduce discomfort after this procedure, we will administer artificial tears (to moisturize the ocular surface) and, if discomfort persists, a local anesthetic.

Photobiomodulation therapy is a non-invasive, short-term procedure in which you will wear a red light mask for 15 minutes. There is a possibility that you will receive a sham treatment, which has no direct therapeutic effect, but your participation is important for the progress of research and may bring future benefits to patients.

Participation in the study will not influence in any way the treatment and medical care to which you are entitled.

5.    FINANCIAL CONSIDERATIONS

Participation in this study does not require any costs on your part, and there will be no financial compensation during the study. The checks required for the study will be performed during routine visits scheduled by your ophthalmologist, with no additional appointments necessary.

6.    CONFIDENTIALITY

All personal data will be kept confidential. The results derived from this study may be published for scientific purposes, but will not include your name or any personal data that could indirectly identify you. The medical information resulting from your participation may be made available to accredited institutions, within the limits imposed by law.

7.    INFORMATION ABOUT OWNERSHIP, USE OF DATA AND BIOLOGICAL SAMPLES

Clinical data and ophthalmological assessment results are collected from the medical record (for which you gave your consent at the time of admission) and are anonymized by assigning a unique code to each participant, without including names or other directly identifiable data. The biological sample (tear film strip) is stored in a freezer, with only the registration number assigned to each patient written on each test tube. The samples collected will be kept for 2 years and then destroyed. The list of correspondence between codes and patient identities is kept separately in a secure file accessible only to the principal investigator. Access to anonymized data is restricted to authorized members of the research team (principal investigator, study coordinator). All data is stored electronically under secure conditions, in accordance with personal data protection legislation.

If photographs of the eye surface are used, they are taken during the consultation and are limited to the area between the eyelids (interpalpebral) or periocular. Depending on the extent of the changes, the photographs will be cropped so that you cannot be identified.

I undertake to comply with the legislation in force regarding the ownership, use, and protection of personal data—GDPR (Regulation 679/2016)—for all study participants.

8. WITHDRAWAL FROM THE STUDY

You are free to decide whether or not you wish to participate in this study.

You are free to withdraw from this study at any time without affecting the medical care required for your illness, by notifying the investigator and signing a separate withdrawal form. However, after the biological samples have been processed, your medical information will remain in the possession of the study team, without you being involved in the subsequent stages.

9.    CONTACT PERSON DURING THE STUDY:

Dr. Timofte (Timofte Zorilă) Mihaela Mădălina

Phone: 0733082205

Email: madalinatim@yahoo.com

10.    AUTHORIZATION

- I have been informed about the objectives of the study and the procedures involved in my participation in the study.
- I had the opportunity to discuss the study and ask questions.
- I agree to participate in the study and am aware that my participation is entirely voluntary.
- I understand that I can withdraw from the study at any time without this affecting the medical care I will receive.

**Investigator**

I explained the nature of the study and the risks involved in participating in the study to the patient. I answered questions and will remain available to the patient for any questions that may arise during the study.

11. LEGISLATIVE ASPECTS

- Decision No. 2/24.10.2017 on the adoption of the Guide on good practice in clinical trials.
- Decision No. 40/27.10.2006 on the approval of the Guide on general considerations
- regarding clinical trials
- Law No. 206 of May 27, 2004 on good conduct in scientific research, technological development, and innovation. (updated in 2016)
- Law No. 17 of February 22, 2001 on the ratification of the European Convention for the Protection of Human Rights and Dignity of the Human Being with regard to the Application of Biology and Medicine.
- Law 319/2003 on the Status of Research and Development Personnel.
- Decision No. 679/2016 on the protection of individuals with regard to the processing of personal data and on the free movement of such data and repealing Directive 95/46/EC (Data Protection Regulation).

1. **References**

1. Day, A. C., Donachie, P. H. J., Sparrow, J. M. & Johnston, R. L. The Royal College of Ophthalmologists’ National Ophthalmology Database study of cataract surgery: Report 1, visual outcomes and complications. *Eye (Basingstoke)* **29**, 552–560 (2015).

2. Naderi, K., Gormley, J. & O’Brart, D. Cataract surgery and dry eye disease: A review. *European Journal of Ophthalmology* vol. 30 840–855 Preprint at https://doi.org/10.1177/1120672120929958 (2020).

3. Sutu, C., Fukuoka, H. & Afshari, N. A. Mechanisms and management of dry eye in cataract surgery patients. *Current Opinion in Ophthalmology* vol. 27 24–30 Preprint at https://doi.org/10.1097/ICU.0000000000000227 (2016).

4. Zhao, L. *et al.* Clinical Characteristic and Tear Film Biomarkers After Myopic FS-LASIK: 1-Year Prospective Follow-up. *Journal of Refractive Surgery* **40**, e508–e519 (2024).

5. Hamblin, M. R. Photobiomodulation or low-level laser therapy. *Journal of Biophotonics* vol. 9 1122–1124 Preprint at https://doi.org/10.1002/jbio.201670113 (2016).

6. Anders, J. J., Arany, P. R., Baxter, G. D. & Lanzafame, R. J. Light-emitting diode therapy and low-level light therapy are photobiomodulation therapy. *Photobiomodulation, Photomedicine, and Laser Surgery* vol. 37 63–65 Preprint at https://doi.org/10.1089/photob.2018.4600 (2019).

7. Hamblin, M. R. Photobiomodulation or low-level laser therapy. *Journal of Biophotonics* vol. 9 1122–1124 Preprint at https://doi.org/10.1002/jbio.201670113 (2016).

8. Yadav, A. & Gupta, A. Noninvasive red and near-infrared wavelength-induced photobiomodulation: promoting impaired cutaneous wound healing. *Photodermatology Photoimmunology and Photomedicine* vol. 33 4–13 Preprint at https://doi.org/10.1111/phpp.12282 (2017).

9. Cannas, C. *et al.* Current Applications and Future Perspectives of Photobiomodulation in Ocular Diseases: A Narrative Review. *Applied Sciences (Switzerland)* vol. 14 Preprint at https://doi.org/10.3390/app14062623 (2024).

10. De Freitas, L. F. & Hamblin, M. R. Proposed Mechanisms of Photobiomodulation or Low-Level Light Therapy. *IEEE Journal of Selected Topics in Quantum Electronics* **22**, 348–364 (2016).

11. Viggiano, P., Boscia, G., Clemente, A. & Giannaccare, G. Photobiomodulation-induced choriocapillaris perfusion enhancement and outer retinal remodelling in intermediate age-related macular degeneration: a promising therapeutic approach with short-term results. *Eye* **39**, 2057–2063 (2025).

12. Asbell, P. A., Maguire, M. G., Peskin, E., Bunya, V. Y. & Kuklinski, E. J. Dry Eye Assessment and Management (DREAM©) Study: Study design and baseline characteristics. *Contemp. Clin. Trials* **71**, 70–79 (2018).

13. Wolffsohn, J. S. *et al.* TFOS DEWS II Diagnostic Methodology report. *Ocular Surface* **15**, 539–574 (2017).

14. Miller, K. L. *et al.* Minimal Clinically Important Difference for the Ocular Surface Disease Index. *Archives of Ophthalmology* **128**, 94–101 (2010).

15. Hopewell, S. *et al.* CONSORT 2025 statement: updated guideline for reporting randomised trials. *BMJ* **389**, (2025).

16. Wolffsohn, J. S. *et al.* TFOS DEWS III: Diagnostic Methodology. *Am. J. Ophthalmol.* **279**, 387–450 (2025).

17. Timofte-Zorila, M. M. *et al.* Novel Tear Biomarkers in Ocular Graft Versus Host Disease Associated with Th1/Th2 Immune Responses: A Case Series and Literature Review. *Int. J. Mol. Sci.* **26**, (2025).

18. Ogata, F. T., Verma, S., Coulson-Thomas, V. J. & Gesteira, T. F. TGF-β-Based Therapies for Treating Ocular Surface Disorders. *Cells* **13**, (2024).

19. Luan, H. H. *et al.* GDF15 Is an Inflammation-Induced Central Mediator of Tissue Tolerance. *Cell* **178**, 1231-1244.e11 (2019).

20. Wang, S. *et al.* GDF-15 Attenuates the Epithelium–Mesenchymal Transition and Alleviates TGFβ2-Induced Lens Opacity. *Transl. Vis. Sci. Technol.* **13**, 2 (2024).

21. Fang, M. *et al.* Functional characteristics of fresh antitumor immune interferer GDF-15 in multiple cancers. *Sci. Rep.* **15**, 30864 (2025).

22. Micera, A. *et al.* Nerve growth factor involvement in the visual system: implications in allergic and neurodegenerative diseases. *Cytokine Growth Factor Rev.* **15**, 411–417 (2004).

23. Nishida, T. Neurotrophic Mediators and Corneal Wound Healing. *Ocul. Surf.* **3**, 194–202 (2005).

24. Kahuam-López, N. *et al.* The Role of Nerve Growth Factor on the Ocular Surface: A Review of the Current Experimental Research and Clinical Practices. *Int. J. Mol. Sci.* **26**, (2025).

25. Sacchetti, M. & Lambiase, A. Neurotrophic factors and corneal nerve regeneration. *Neural Regen. Res.* **12**, (2017).

26. Chen, P.-H., Chen, X. & He, X. Platelet-derived growth factors and their receptors: Structural and functional perspectives. *Biochimica et Biophysica Acta (BBA) - Proteins and Proteomics* **1834**, 2176–2186 (2013).

27. Lee, C., Zhang, F., Tang, Z., Liu, Y. & Li, X. PDGF-C: a new performer in the neurovascular interplay. *Trends Mol. Med.* **19**, 474–486 (2013).

28. Li, X. *et al.* *VEGF-Independent Angiogenic Pathways Induced by PDGF-C*. *Oncotarget* vol. 1 www.impactjournals.com/oncotarget/www.impactjournals.com/oncotarget (2010).

29. Klenkler, B., Sheardown, H. & Jones, L. Growth Factors in the Tear Film: Role in Tissue Maintenance, Wound Healing, and Ocular Pathology. *Ocul. Surf.* **5**, 228–239 (2007).

30. Zhao, Y. *et al.* Corneal subbasal nerve alterations and tear cytokine associations in ocular chronic graft-versus-host disease. *Front. Med. (Lausanne).* **12**, (2025).

31. HOCK, J. M., RAISZ, L. G. & CANALIS, E. Parathyroid Hormone. *The Parathyroids* 183–198 (2001) doi:10.1016/B978-012098651-4/50013-4.

32. Tumia, N. S. & Johnstone, A. J. Platelet derived growth factor-AB enhances knee meniscal cell activity in vitro. *Knee* **16**, 73–76 (2009).

33. Dimmeler, S. Platelet-Derived Growth Factor CC — A Clinically Useful Angiogenic Factor at Last? *New England Journal of Medicine* **352**, 1815–1816 (2005).

34. Martínez, C. E., Smith, P. C. & Palma Alvarado, V. A. The influence of platelet-derived products on angiogenesis and tissue repair: a concise update. *Front. Physiol.* **Volume 6-2015**, (2015).

35. Antwi, A., Schill, A. W., Redfern, R. & Ritchey, E. R. Effect of low-level light therapy in individuals with dry eye disease. *Ophthalmic and Physiological Optics* **44**, 1464–1471 (2024).

36. Lang, F., Li, Y., Yao, R. & Jiang, M. Osteopontin in Chronic Inflammatory Diseases: Mechanisms, Biomarker Potential, and Therapeutic Strategies. *Biology (Basel).* **14**, (2025).

37. Lund, S. A., Giachelli, C. M. & Scatena, M. The role of osteopontin in inflammatory processes. *J. Cell Commun. Signal.* **3**, 311–322 (2009).

38. Kim, J. *et al.* Osteopontin is a biomarker for early autoimmune uveoretinitis. *Neural Regen. Res.* **17**, (2022).

39. Mori, R., Shaw, T. J. & Martin, P. Molecular mechanisms linking wound inflammation and fibrosis: knockdown of osteopontin leads to rapid repair and reduced scarring. *Journal of Experimental Medicine* **205**, 43–51 (2008).

40. Lekwuwa, M., Choudhary, M., Lad, E. M. & Malek, G. Osteopontin accumulates in basal deposits of human eyes with age-related macular degeneration and may serve as a biomarker of aging. *Modern Pathology* **35**, 165–176 (2022).

41. Dekker, M. *et al.* High levels of osteoprotegerin are associated with coronary artery calcification in patients suspected of a chronic coronary syndrome. *Sci. Rep.* **11**, 18946 (2021).

42. Caidahl, K., Ueland, T. & Aukrust, P. Osteoprotegerin: A Biomarker With Many Faces. *Arterioscler. Thromb. Vasc. Biol.* **30**, 1684–1686 (2010).

43. Greenhill, C. Osteoprotegerin sources examined. *Nat. Rev. Endocrinol.* **16**, 678 (2020).

44. Walsh, M. C. & Choi, Y. Biology of the RANKL-RANK-OPG system in immunity, bone, and beyond. *Frontiers in Immunology* vol. 5 Preprint at https://doi.org/10.3389/fimmu.2014.00511 (2014).

45. Pritzker, L. B., Scatena, M. & Giachelli, C. M. The Role of Osteoprotegerin and Tumor Necrosis Factor-related Apoptosis-inducing Ligand in Human Microvascular Endothelial Cell Survival. *Mol. Biol. Cell* **15**, 2834–2841 (2004).

46. Benslimane-Ahmim, Z. *et al.* Osteoprotegerin, a new actor in vasculogenesis, stimulates endothelial colony-forming cells properties. *Journal of Thrombosis and Haemostasis* **9**, 834–843 (2011).

47. Abu El-Asrar, A. M. *et al.* Osteoprotegerin is a new regulator of inflammation and angiogenesis in proliferative diabetic retinopathy. *Invest. Ophthalmol. Vis. Sci.* **58**, 3189–3201 (2017).

48. Abu El-Asrar, A. M. *et al.* Unbalanced Vitreous Levels of Osteoprotegerin, RANKL, RANK, and TRAIL in Proliferative Diabetic Retinopathy. *Ocul. Immunol. Inflamm.* **26**, 1248–1260 (2018).

49. Dekker, M. *et al.* High levels of osteoprotegerin are associated with coronary artery calcification in patients suspected of a chronic coronary syndrome. *Sci. Rep.* **11**, 18946 (2021).

50. Martinez-Gutierrez, A. *et al.* Hyaluronic and Succinic Acid: New Biostimulating Combination to Counteract Dermal and Subcutaneous Aging. *Int. J. Mol. Sci.* **26**, (2025).

51. Panigrahi, T. *et al.* Trehalose augments autophagy to mitigate stress induced inflammation in human corneal cells. *Ocular Surface* **17**, 699–713 (2019).

52. Timofte-Zorila, M.-M. *et al.* Effect of Low-Level Light Therapy on Ocular Surface Parameters in Patients Undergoing Cataract Surgery: A Prospective Double-Masked Randomized Controlled Clinical Trial. *Ophthalmol. Ther.* **14**, 2557–2569 (2025).

53. Giannaccare, G. *et al.* Outcomes of low-level light therapy before and after cataract surgery for the prophylaxis of postoperative dry eye: a prospective randomised double-masked controlled clinical trial. *British Journal of Ophthalmology* **108**, 1172 (2024).
